# Inflammatory monocytes mediate control of acute alphavirus infection in mice

**DOI:** 10.1371/journal.ppat.1006748

**Published:** 2017-12-15

**Authors:** Kelsey C. Haist, Kristina S. Burrack, Bennett J. Davenport, Thomas E. Morrison

**Affiliations:** Department of Immunology and Microbiology, University of Colorado School of Medicine, Aurora, Colorado, United States of America; University of Pennsylvania School of Medicine, UNITED STATES

## Abstract

Chikungunya virus (CHIKV) and Ross River virus (RRV) are mosquito-transmitted alphaviruses that cause debilitating acute and chronic musculoskeletal disease. Monocytes are implicated in the pathogenesis of these infections; however, their specific roles are not well defined. To investigate the role of inflammatory Ly6C^hi^CCR2^+^ monocytes in alphavirus pathogenesis, we used CCR2-DTR transgenic mice, enabling depletion of these cells by administration of diptheria toxin (DT). DT-treated CCR2-DTR mice displayed more severe disease following CHIKV and RRV infection and had fewer Ly6C^hi^ monocytes and NK cells in circulation and muscle tissue compared with DT-treated WT mice. Furthermore, depletion of CCR2^+^ or Gr1^+^ cells, but not NK cells or neutrophils alone, restored virulence and increased viral loads in mice infected with an RRV strain encoding attenuating mutations in nsP1 to levels detected in monocyte-depleted mice infected with fully virulent RRV. Disease severity and viral loads also were increased in DT-treated CCR2-DTR^+^;*Rag1*^-/-^ mice infected with the nsP1 mutant virus, confirming that these effects are independent of adaptive immunity. Monocytes and macrophages sorted from muscle tissue of RRV-infected mice were viral RNA positive and had elevated expression of *Irf7*, and co-culture of Ly6C^hi^ monocytes with RRV-infected cells resulted in induction of type I IFN gene expression in monocytes that was *Irf3*;*Irf7* and *Mavs*-dependent. Consistent with these data, viral loads of the attenuated nsP1 mutant virus were equivalent to those of WT RRV in *Mavs*^-/-^ mice. Finally, reconstitution of *Irf3*^*-/-*^;*Irf7*^-/-^ mice with CCR2-DTR bone marrow rescued mice from severe infection, and this effect was reversed by depletion of CCR2^+^ cells, indicating that CCR2^+^ hematopoietic cells are capable of inducing an antiviral response. Collectively, these data suggest that MAVS-dependent production of type I IFN by monocytes is critical for control of acute alphavirus infection and that determinants in nsP1, the viral RNA capping protein, counteract this response.

## Author summary

Mosquito-transmitted arthritogenic alphaviruses, such as chikungunya virus (CHIKV), Mayaro virus, and Ross River virus (RRV), cause large disease outbreaks. Infection with these viruses results in severe pain and inflammation in joints, tendons, and muscles, likely due to direct viral infection of these tissues, that can persist for years. Monocytes and macrophages have been implicated in the damaging effects of the inflammation, however, the role of these cell types in control of alphaviral infection are poorly understood. Using mouse models and an attenuated RRV with mutations in the nsP1 gene, we found that monocytes are critical to control acute infection and to reduce disease severity. Furthermore, we found that monocytes respond to virus-infected cells by increasing expression levels of type I interferon, a critical antiviral defense system. The induction of type I interferon in monocytes was dependent on MAVS, a signaling protein downstream of cytosolic viral RNA sensor proteins. Similar to monocytes, MAVS was required to control infection with the nsP1 mutant RRV. These studies suggest that monocytes control acute alphavirus infection and that determinants in nsP1, the viral RNA capping protein, counteract this response. Thus, therapeutic strategies targeting these cells for the treatment of these viral inflammatory diseases should do so without compromising their role in innate immunity.

## Introduction

Chikungunya (CHIKV), Ross River (RRV), Mayaro, and o’nyong-nyong viruses are mosquito-transmitted positive-sense, enveloped RNA viruses in the *Alphavirus* genus of the family *Togaviridae*. These viruses cause both endemic and explosive epidemics of debilitating acute and chronic musculoskeletal disease [1]. CHIKV has caused epidemics of unprecedented scale within the past decade in many regions of the world. Beginning in 2004, CHIKV re-emerged from Africa and spread to islands in the Indian Ocean, India, and countries in Southeast Asia, the South Pacific, and Europe [2]. In October of 2013, local transmission of CHIKV occurred on the Caribbean island of Saint Martin [3]. Since that time, the virus has spread throughout much of the Americas resulting in approximately two-million confirmed and suspected cases in 45 countries and territories [4, 5].

CHIKV and RRV infection typically present with a sudden onset of high fever and severe pain and inflammation in peripheral joints [1, 6]. This acute phase lasts for 1 to 2 weeks and often resolves without further complications. However, some disease signs and symptoms, such as joint swelling, joint stiffness, arthralgia, arthritis, and tendonitis/tenosynovitis, can last for months to years in 10–50% of patients [7–13].

The injury and inflammation of musculoskeletal tissues observed during infection with arthritogenic alphaviruses is likely initiated by direct infection of these tissues. In humans and experimentally-infected mice, viral infection of musculoskeletal tissues promotes a robust host inflammatory response, some aspects of which have been directly implicated in the musculoskeletal tissue damage [14–24]. For example, musculoskeletal tissues of CHIKV- or RRV-infected humans and mice are heavily infiltrated with monocytes and macrophages [8, 25–32], and several studies suggest that these cell populations promote the pathogenesis of acute CHIKV and RRV infection. In mice, treatment with macrophage toxic agents such as silica [27] or clodronate-loaded liposomes [19, 25] prior to CHIKV or RRV infection resulted in less severe acute virus-induced disease. Furthermore, treatment of RRV-infected mice with methotrexate accelerated the development of musculoskeletal disease signs, and this enhancement was associated with an increased influx of monocytes into musculoskeletal tissues [33]. Monocyte chemoattractant protein-1 (MCP-1), or CCL2, a chemokine responsible for monocyte egress from the bone marrow and migration into certain tissues within inflammatory contexts [34–38], is elevated during CHIKV and RRV infection of humans and mice [19, 25, 39–46]. Treatment of mice with bindarit, a small molecule that inhibits production of MCP-1 and other MCPs, reduced the severity of both CHIKV- and RRV-induced disease signs [46–48]. Collectively, these findings support a pathogenic role for monocytes and macrophages during CHIKV and RRV infection. However, some studies also have suggested a protective role for these cell populations. Administration of clodronate-loaded liposomes to mice prolonged viremia following CHIKV infection [25], suggesting that monocytes, macrophages, and/or other phagocytic cells contribute to control of virus infection. In addition, CHIKV infection of *Ccr2*^-/-^ mice resulted in more severe arthritic disease characterized by an absence of monocytes and macrophages and increased numbers of neutrophils in musculoskeletal tissues [14], suggesting that monocytes protect from more severe forms of joint pathology.

Despite this robust host inflammatory response, the immunological mechanisms that mediate control of acute alphavirus infection in musculoskeletal tissues are not well defined. One component of the innate immune response to arthritogenic alphavirus infection, the type I interferon (IFN) response, is essential for control of these infections, as mice with defects in type I IFN signaling succumb rapidly to CHIKV and RRV infection [49–54]. Although studies indicate that expression of the receptor for type I IFN on nonhematopoietic cells is essential for control of CHIKV infection [49], the cellular sources of type I IFN are less clear. Experiments in bone marrow chimeric mice indicate that hematopoietic cell *Irf3*;*Irf7* and *Tlr3*, signaling molecules that when activated promote type I IFN expression, mediate protection against CHIKV infection [51, 55], suggesting that hematopoietic cells are one important source of type I IFN during alphavirus infection. Consistent with these data, exposure of human monocytes to CHIKV results in the production of type I IFNs [56, 57].

In previous studies, we identified an attenuated strain of RRV (DC5692) that in contrast to the virulent RRV-T48 strain does not cause severe musculoskeletal disease in mice [58]. We used these two strains in chimeric virus studies to define viral virulence determinants [54, 58]. Through this work, we identified polymorphisms in nsP1 that, when introduced into the RRV-T48 genome, enhanced RRV-T48 sensitivity to type I IFN and attenuated RRV-T48 infection in WT and *Rag1*^-/-^ mice [54, 58]. The attenuation of this nsP1 mutant virus (RRV-T48-nsP1^6M^) in both WT and *Rag1*^−/−^ mice correlated with reduced viral loads in skeletal muscle tissue at the time point this tissue is infiltrated with inflammatory monocytes [54]. In this study, we used CCR2-DTR mice [59], which express the diphtheria toxin receptor (DTR) under control of the CCR2 promoter, to further investigate the role of inflammatory monocytes during acute alphavirus infection. Depletion of CCR2^+^ inflammatory monocytes resulted in increased disease severity and viral burdens in mice infected with the nsP1 mutant virus as well as in mice infected with CHIKV, suggesting that Ly6C^hi^ monocytes protect from alphavirus infection. Type I IFN gene expression was induced in monocytes co-cultured with CHIKV- or RRV-infected cells in an *Irf3*;*Irf7*- and *Mavs*-dependent manner, indicating that monocytes express type I IFN in response to alphavirus infection or alphavirus-infected cells. Consistent with these data, monocytes and macrophages sorted from skeletal muscle tissue of RRV-infected mice were positive for viral RNA and had elevated expression levels of *Irf7* mRNA, a transcription factor that promotes type I IFN production. Similar to mice depleted of Ly6C^hi^ monocytes, viral loads in the muscle tissue of *Mavs*-deficient mice infected with WT RRV or the nsP1 mutant virus were equivalent. Furthermore, reconstitution of *Irf3*^*-/-*^;*Irf7*^-/-^ mice with CCR2-DTR bone marrow rescued mice from severe infection, and this effect was reversed by depletion of CCR2^+^ cells, indicating that CCR2^+^ hematopoietic cells are capable of inducing an antiviral response. These studies suggest that Ly6C^hi^ inflammatory monocytes contribute to control of acute alphavirus infection by a MAVS-and IRF3;IRF7-dependent mechanism.

## Results

### Depletion of Ly6C^hi^ monocytes and natural killer (NK) cells in DT-treated CCR2-DTR mice

**Fig 1:**
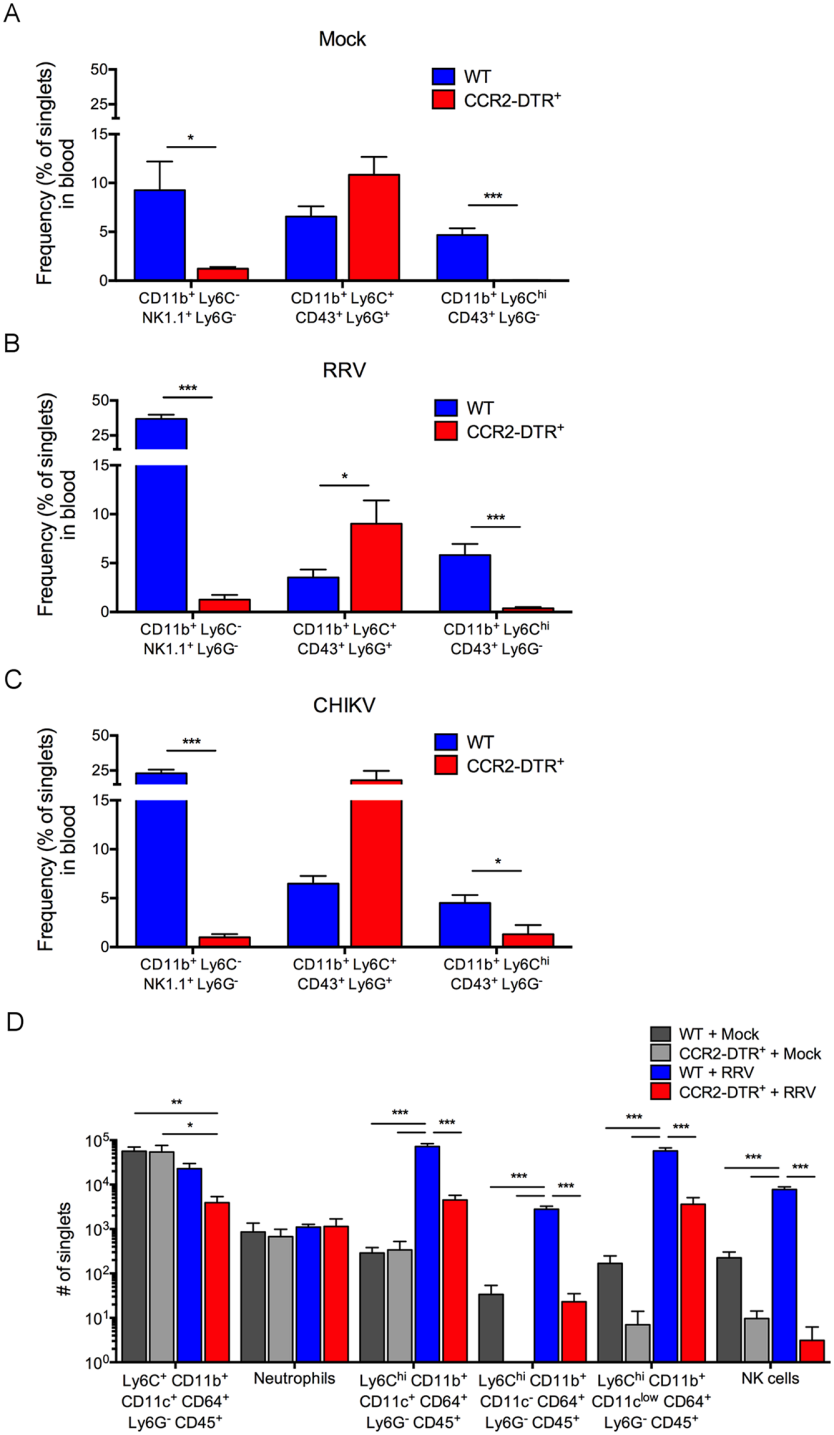
Ly6C^hi^ monocytes and NK cells are depleted from DT-treated CCR2-DTR mice. (A, B, C) WT (n = 6–7 mice/group) or CCR2-DTR (n = 5–6 mice/group) C57BL/6 mice were inoculated in the left rear footpad with (A) PBS, (B) RRV-T48, or (C) CHIKV. At days -1 and +2 relative to infection, mice were i.p. administered DT. 24 h after the last DT administration, the frequency of Ly6C^hi^ monocytes (Ly6C^hi^CD11b^+^CD43^+^Ly6G^-^), NK cells (NK1.1^+^CD11b^+^Ly6C^-^Ly6G^-^), and neutrophils (Ly6G^+^CD11b^+^CD43^+^Ly6C^+^) in the blood were determined by flow cytometry. Data are pooled from two independent experiments. *P* values were determined by Student’s t-test. *, *P* < 0.05; ***, *P* < 0.001. (D) WT (n = 4–5 mice/group) or CCR2-DTR (n = 2–5 mice/group) C57BL/6 mice were inoculated in the left rear footpad with either PBS or RRV-T48. DT was administered as described for A-C. At 48 h after the last DT administration, the number of NK cells (NK1.1^+^CD11b^+^Ly6C^-^Ly6G^-^), neutrophils (Ly6G^+^CD11b^+^CD43^+^Ly6C^+^), and various Ly6C^+^CD11b^+^ and Ly6C^hi^CD11b^+^ myeloid subsets were determined by flow cytometry. Data are pooled from two independent experiments. *P* values were determined by one-way ANOVA with a Tukey’s multiple comparisons test. *, *P* < 0.05; **, *P* < 0.01; ***, *P* < 0.001.

In previous studies, we identified mutations in nsP1 that attenuated RRV-T48 infection in WT and *Rag1*^-/-^ mice [54, 58]. The attenuation of the nsP1 mutant virus (RRV-T48-nsP1^6M^) in both WT and *Rag1*^−/−^ mice correlated with reduced viral loads in skeletal muscle tissue at the time point (5 dpi) this tissue is infiltrated with NK cells and inflammatory monocytes [54]. However, depletion of NK cells did not restore viral loads in mice infected with RRV-T48-nsP1^6M^ [54], suggesting that the mutations in nsP1 render the virus more susceptible to monocyte-mediated antiviral defenses. Murine Ly6C^hi^ inflammatory monocytes express high levels of CCR2, a chemokine receptor that promotes emigration from the bone marrow [38, 60]. To investigate the role of Ly6C^hi^ inflammatory monocytes in control of RRV-T48-nsP1^6M^ infection, we used transgenic CCR2 depleter mice in which the CCR2 locus was modified to place a simian diphtheria toxin receptor (DTR) and enhanced cyan fluorescent protein (CFP), separated by an aphthovirus 2A cleavage site, under control of the endogenous CCR2 promoter (CCR2-DTR) [59]. These mice have been used in numerous studies for efficient *in vivo* depletion of inflammatory monocytes and derivative cells [59, 61–63]. To confirm these data in our laboratory, CCR2-DTR mice and littermate wild-type (WT) control mice were administered diphtheria toxin (DT) by intraperitoneal (i.p.) injection one day prior and 2 days after either mock-inoculation or inoculation with RRV-T48 or CHIKV. At 24 hours (h) after the last DT administration, we measured the frequency of various cell populations in the blood by flow cytometry. Similar to other studies [59, 61], in mock-, RRV-, and CHIKV-inoculated CCR2-DTR mice, we detected depletion of both Ly6C^hi^ monocytes (Ly6C^hi^CD11b^+^CD43^+^Ly6G^-^) and NK cells (NK1.1^+^CD11b^+^Ly6C^-^Ly6G^-^) in comparison with DT-treated WT mice (Fig 1 and S1 Fig). In contrast, the frequency of neutrophils (Ly6G^+^CD11b^+^CD43^+^Ly6C^+^) in the blood of DT-treated CCR2-DTR mice was increased compared with WT mice, indicating that this cell population was not deleted by DT treatment. Consistent with these data, Ly6C^hi^ monocytes and NK cells in the blood of CCR2-DTR mice were CFP^+^, but neutrophils lacked CFP expression (S2 Fig).

In addition to the circulation, we also assessed the impact of DT administration on cell populations in the muscle tissue of control and RRV-T48-infected mice (Fig 1D and S3 Fig). At 48 h after the last DT administration, we quantified leukocytes in enzymatically-digested muscle tissue by flow cytometry. Muscle tissue from mock-infected mice had low numbers of NK cells and Ly6C^hi^CD11b^+^ cells that were depleted in DT-treated CCR2-DTR mice. We also detected a population of Ly6C^+^CD11b^+^CD11c^+^CD64^+^Ly6G^-^ cells that was unaffected in DT-treated CCR2-DTR mice (Fig 1D and S3 Fig). NK cells and Ly6C^hi^ cell populations were increased in muscle tissue of RRV-T48-infected mice, and these cells were depleted in RRV-T48-infected CCR2-DTR mice (Fig 1D and S3 Fig). The Ly6C^+^CD11b^+^CD11c^+^CD64^+^Ly6G^-^ cell population found in muscle tissue of mock-infected mice also was unaffected following DT treatment of RRV-T48-infected CCR2-DTR mice (Fig 1D and S3 Fig). These data indicate that DT treatment of CCR2-DTR mice reduces accumulation of NK cells and Ly6C^hi^ monocytes in muscle tissue.

### Depletion of CCR2^+^ cells results in more severe RRV and CHIKV disease and increased viral burdens in tissues

**Fig 2:**
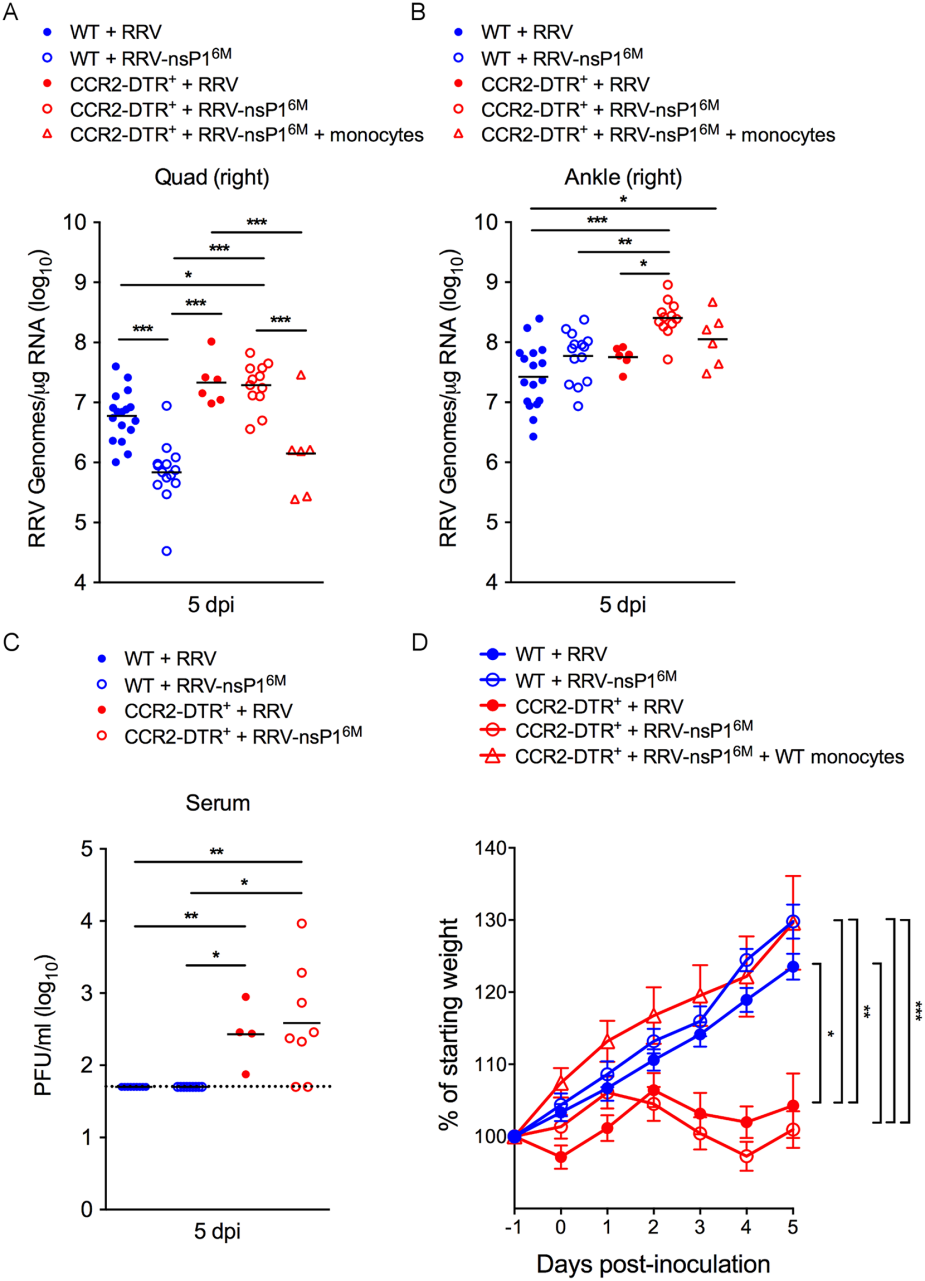
Depletion of CCR2^+^ cells enhanced viral loads in tissues and the severity of acute RRV infection. WT or CCR2-DTR C57BL/6 mice were inoculated in the left rear footpad with 1,000 PFU of RRV-T48 or RRV-T48-nsP1^6M^. At days -1 and +2 relative to infection, mice were i.p. administered DT. 1 h prior to virus infection, Ly6C^hi^ monocytes were adoptively transferred into a subset of CCR2-DTR mice. At 5 dpi, viral RNA levels in (A) skeletal muscle tissue and (B) contralateral ankle tissue were quantified by qRT-PCR. (C) At 5 dpi, infectious virus in the serum was quantified by plaque assay. (D) The percent starting body weight was determined daily (n = 6–17 mice/group). Data are pooled from three independent experiments. *P* values were determined by one-way ANOVA with a Tukey’s multiple comparison test (A-C) and a repeated measures two-way ANOVA with a Bonferroni’s multiple comparison test (D). *, *P* <0.05; **, *P* < 0.01; ***, *P* < 0.001.

To assess whether depletion of CCR2^+^ cells rescues viral loads of the attenuated nsP1 mutant virus, WT and CCR2-DTR mice were administered DT as described above, inoculated with RRV-T48 or RRV-T48-nsP1^6M^, and viral loads in skeletal muscle tissue at 5 dpi were quantified by qRT-PCR. Consistent with our previous findings [54], viral RNA levels in skeletal muscle tissue of RRV-T48-nsP1^6M^-infected WT mice at 5 dpi were reduced compared with RRV-T48-infected mice (9-fold; *P*, < 0.001) (Fig 2A). Compared with viral RNA levels in RRV-T48-nsP1^6M^-infected WT mice, viral RNA levels in the skeletal muscle tissue of RRV-T48-nsP1^6M^-infected CCR2-DTR mice were increased (28-fold; *P*, < 0.001) (Fig 2A) and were similar to viral RNA levels detected in the skeletal muscle tissue of RRV-T48-infected CCR2-DTR mice (Fig 2A). Similarly, the amount of viral RNA in the ankle joint of RRV-T48-nsP1^6M^-infected CCR2-DTR mice was increased (4-fold; *P*, < 0.001) compared with the amount of viral RNA in the ankle of RRV-T48-nsP1^6M^-infected WT mice (Fig 2B), suggesting that the nsP1 mutant virus also is more sensitive to monocyte-mediated control in this tissue. CCR2-DTR mice infected with either virus also had increased viral loads in the serum at this time point (Fig 2C), further supporting an important role for CCR2^+^ cells in the control of RRV infection. In addition, RRV-T48 and RRV-T48-nsP1^6M^-infected CCR2-DTR mice displayed a marked defect in weight gain over the course of 5 days (Fig 2D). Importantly, this effect was specific to RRV-infected DT-treated CCR2-DTR mice, as age-matched naïve CCR2-DTR mice administered DT in a similar manner did not exhibit the same defect in weight gain (S4 Fig). To confirm a role for inflammatory monocytes in protection, enriched bone marrow Ly6C^hi^ monocytes (S5 Fig) were adoptively transferred into DT-treated CCR2-DTR mice 1 h prior to infection with RRV-T48-nsP1^6M^. As shown in Fig 2A, viral RNA levels in skeletal muscle tissue at 5 dpi in these mice were similar to the viral RNA levels detected in RRV-T48-nsP1^6M^-infected WT mice. In addition, adoptive transfer of enriched bone marrow Ly6C^hi^ monocytes into DT-treated CCR2-DTR mice reversed the marked defect in weight gain observed in RRV-T48-nsP1^6M^-infected CCR2-DTR mice that did not receive monocytes (Fig 2D).

To evaluate whether the effects of depletion of CCR2^+^ cells on RRV infection are independent of T or B cells, we bred the CCR2-DTR transgene onto the *Rag1*^-/-^ C57BL/6 background. *Rag1*^-/-^ mice with or without the CCR2-DTR allele were treated with DT in the same manner and assessed at 5 dpi for effects on viral loads in skeletal muscle tissue and for effects on weight gain. Consistent with our previous findings [54], viral RNA levels in skeletal muscle tissue of RRV-T48-nsP1^6M^-infected *Rag1*^-/-^ mice at 5 dpi were reduced compared with RRV-T48-infected *Rag1*^-/-^ mice (10-fold; *P*, < 0.001) (S6 Fig). Similar to *Rag1*-sufficient CCR2-DTR mice, viral RNA levels in the skeletal muscle tissue of RRV-T48-nsP1^6M^-infected *Rag1*^-/-^;CCR2-DTR mice were elevated compared with viral RNA levels in the skeletal muscle tissue of RRV-T48-nsP1^6M^-infected *Rag1*^-/-^ littermates (8-fold; *P*, < 0.001) (S6 Fig) and were similar to viral RNA levels detected in the skeletal muscle tissue of RRV-T48-infected *Rag1*^-/-^ and *Rag1*^-/-^;CCR2-DTR mice (S6 Fig). RRV-T48 and RRV-T48-nsP1^6M^-infected *Rag1*^-/-^;CCR2-DTR mice also displayed a marked defect in weight gain during the 5 day study (S6 Fig). These findings suggest that the effects of depletion of CCR2^+^ cells on disease severity and viral burdens during RRV infection occurs independently of any potential effects, direct or indirect, on T and B cell responses.

**Fig 3:**
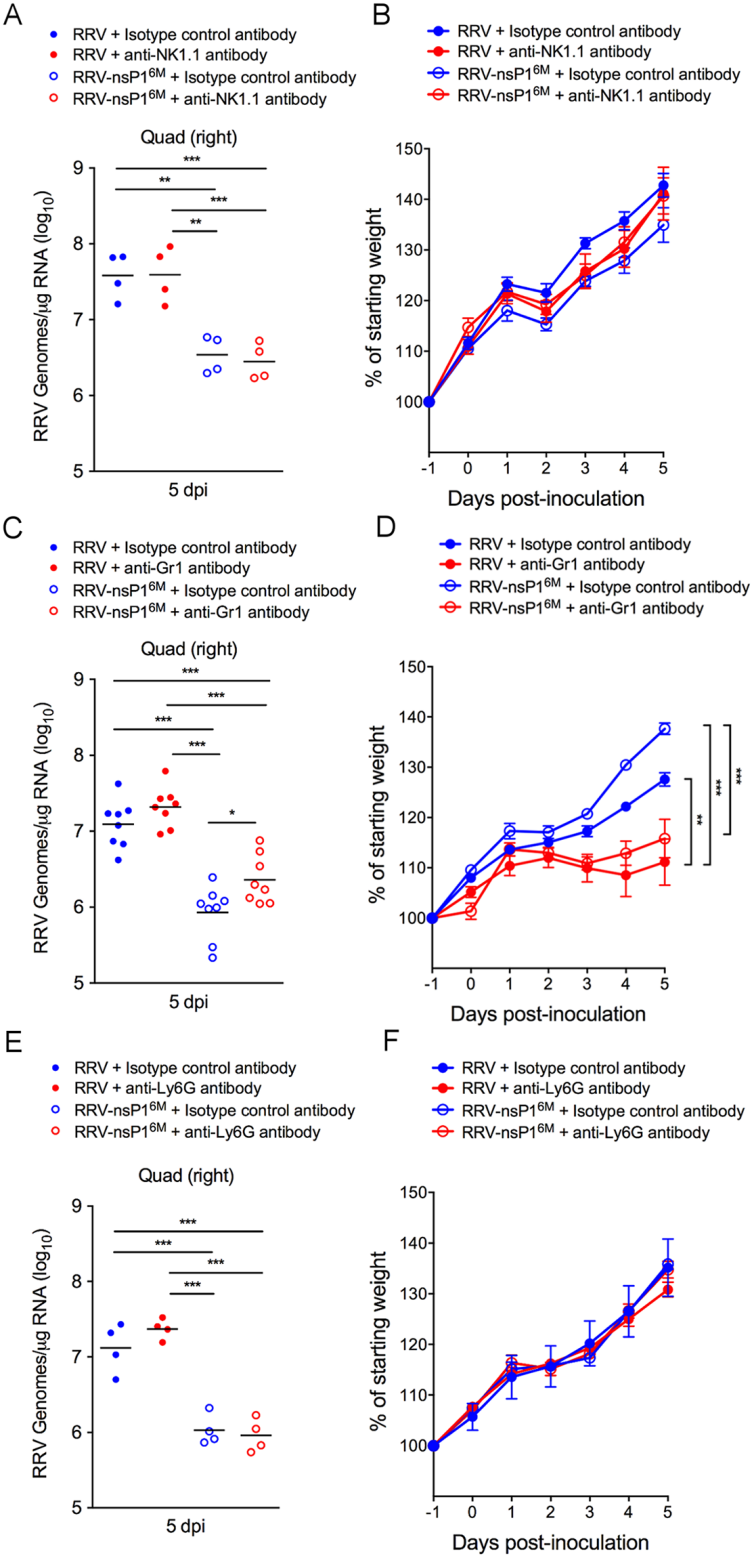
Antibody-mediated depletion of Ly6C^hi^ monocytes exacerbates acute RRV infection. WT C57BL/6 mice were administered (A and B) anti-NK1.1 (n = 4) or a control antibody (n = 4), (C and D) anti-Gr1 (n = 8) or a control antibody (n = 8), or (E and F) anti-Ly6G (n = 4) or a control antibody (n = 4) at day -1 and day +2 relative to infection with the indicated viruses. At 5 dpi, (A, C, E) viral RNA levels in skeletal muscle tissue were quantified by qRT-PCR, and (B, D, F) the percent starting body weight was determined daily. Data are from one (anti-NK1.1 and anti-Ly6G) or two (anti-Gr1) independent experiments. *P* values were determined by one-way ANOVA with a Tukey’s multiple comparison test (A, C, E) or a repeated measures two-way ANOVA with a Bonferroni’s multiple comparison test (B, D, F). *, *P* <0.05; **, *P* < 0.01; ***, *P* < 0.001.

As both Ly6C^hi^ inflammatory monocytes and NK cells are depleted when CCR2-DTR mice are treated with DT (Fig 1), we assessed whether depletion of NK cells alone would enhance viral loads in RRV-T48 or RRV-T48-nsP1^6M^-infected mice. Administration of an anti-NK1.1 depleting antibody at one day prior and two days after virus inoculation effectively depleted NK cells (NK1.1^+^CD11b^+^Ly6C^-^CD3ε^-^) in the blood (S7 Fig) but did not diminish the frequency of Ly6G^+^ neutrophils (Ly6G^+^CD11b^+^CD43^lo^Ly6C^+^) or Ly6C^hi^ monocytes (Ly6C^hi^CD11b^+^CD43^+^Ly6G^-^) in the blood (S7 Fig). Consistent with our previous results [54], depletion of NK cells had no effect on the amount of viral RNA detected in skeletal muscle tissue of RRV-T48- or RRV-T48-nsP1^6M^-infected WT mice (Fig 3A) or weight gain (Fig 3B). In contrast, administration of an anti-Gr1 antibody effectively depleted Ly6G^+^ neutrophils and reduced the frequency of Ly6C^hi^ inflammatory monocytes (S7 Fig). We detected increased viral RNA levels in skeletal muscle tissue of RRV-T48-nsP1^6M^-infected mice treated with the anti-Gr1 antibody compared with control antibody-treated mice (3-fold; *P* < 0.05) (Fig 3C). Similar to DT-treated CCR2-DTR mice, in which we observed depletion of Ly6C^hi^ inflammatory monocytes but not neutrophils (Fig 1), administration of anti-Gr1 antibody resulted in significantly reduced weight gain of RRV-T48 and RRV-T48-nsP1^6M^-infected mice compared with mice treated with a control antibody (Fig 3D). The effects of the anti-Gr1 antibody treatment on viral loads in the skeletal muscle of RRV-T48-nsP1^6M^-infected mice and weight gain was less pronounced than observed in DT-treated CCR2-DTR mice and was associated with a less robust depletion of Ly6C^hi^ inflammatory monocytes (S7 Fig) compared with DT-treated CCR2-DTR mice (Fig 1B), suggesting that a more complete depletion of Ly6C^hi^ inflammatory monocytes is required to fully enhance the pathogenicity of the attenuated RRV-T48-nsP1^6M^ mutant virus. To evaluate the extent to which depletion of neutrophils contributed to the effects of anti-Gr1 antibody treatment on viral loads and weight gain, mice were administered anti-Ly6G antibody. Treatment with anti-Ly6G antibody resulted in robust depletion of neutrophils, but not monocytes or NK cells (S7 Fig). However, anti-Ly6G antibody did not affect viral RNA levels in skeletal muscle tissue (Fig 3E) or weight gain (Fig 3F), suggesting that the effect of anti-Gr1 antibody treatment on viral loads and weight gain was due to the depletion of monocytes. These data further suggest that monocytes play an antiviral role during acute RRV infection.

**Fig 4:**
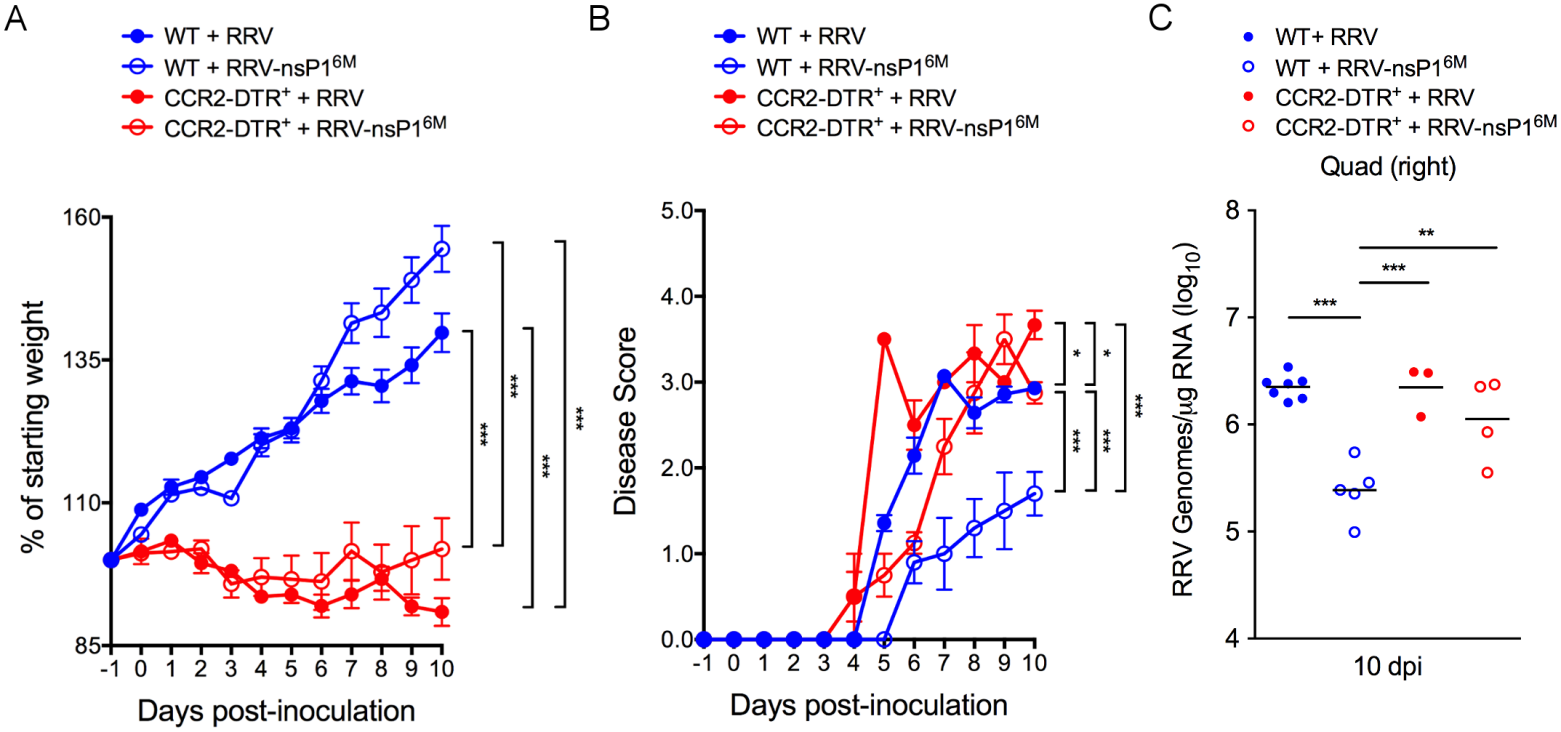
Depletion of CCR2^+^ cells enhances pathogenicity of the attenuated RRV-T48-nsP1^6M^ mutant virus. WT or CCR2-DTR C57BL/6 mice (n = 3–7 mice/group) were inoculated in the left rear footpad with 1,000 PFU of RRV-T48 or RRV-T48-nsP1^6M^. At days -1 and +2 relative to infection, mice were i.p. administered DT. (A) The percent starting body weight and (B) musculoskeletal disease score were determined daily. (C) At 10 dpi, viral RNA levels in skeletal muscle tissue were quantified by qRT-PCR. *P* values were determined by a repeated measures two-way ANOVA with a Bonferroni’s multiple comparison test (A, B) and by one-way ANOVA with a Tukey’s multiple comparison test (C) *, *P* <0.05; **, *P* < 0.01; ***, *P* < 0.001.

We next assessed whether depletion of CCR2^+^ cells affected virus-induced disease severity or viral burdens at later times post-infection. For these experiments, WT and CCR2-DTR mice were administered DT at day -1 and day +2 relative to virus inoculation and mice were monitored for 10 days, the peak of the acute disease phase [54, 58, 64], for effects on weight gain and for the development of virus-induced musculoskeletal disease signs. CCR2-DTR mice infected with either virus displayed reduced weight gain compared with virus-infected WT mice (Fig 4A) and uninfected CCR2-DTR mice administered DT in a similar manner (S4 Fig). In addition, RRV-T48-nsP1^6M^-infected CCR2-DTR mice developed more severe defects in hind limb gripping ability and gait compared with RRV-T48-nsP1^6M^-infected WT mice (Fig 4B), and these disease signs were similar in severity to those detected in RRV-T48-infected WT mice. Furthermore, despite being a full 8 days after the last administration of DT, viral RNA levels in skeletal muscle tissue of RRV-T48-nsP1^6M^-infected CCR2-DTR mice were increased compared with viral RNA levels in skeletal muscle tissue of RRV-T48-nsP1^6M^-infected WT mice (5-fold; *P*, < 0.01) and similar to viral RNA levels detected in mice infected with RRV-T48 (Fig 4C). These data suggest that early depletion of Ly6C^hi^ inflammatory monocytes during RRV infection results in a lasting impact on disease severity and virus control. Collectively, our findings suggest that the nsP1 mutant virus is more sensitive to the antiviral effects of Ly6C^hi^ inflammatory monocytes, and that in the absence of these cells this mutant virus has a similar capacity to infect and cause disease as the WT virus.

**Fig 5:**
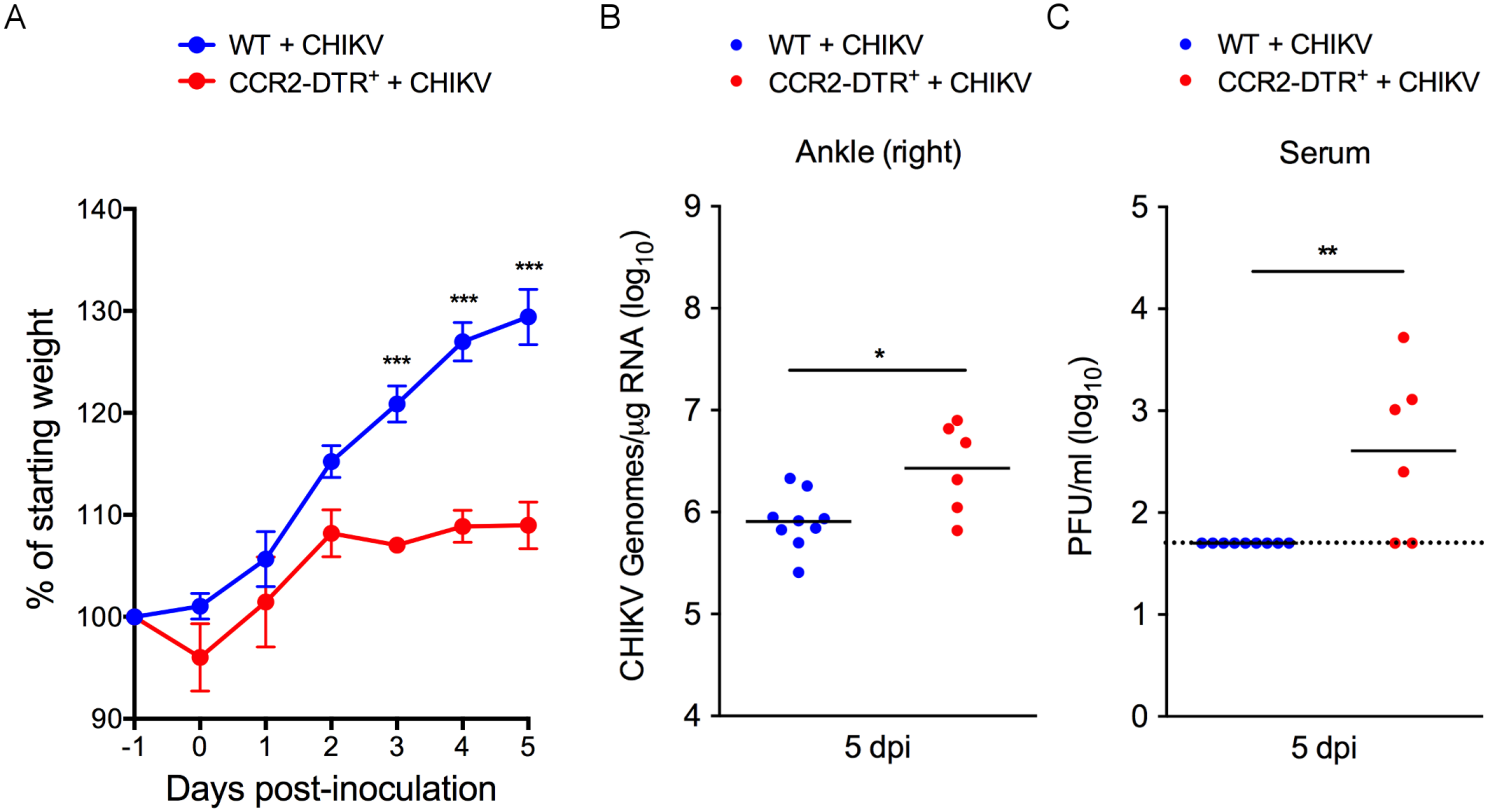
Depletion of CCR2^+^ cells enhanced viral loads in tissues and the severity of disease during acute CHIKV infection. WT (n = 9) or CCR2-DTR (n = 6) C57BL/6 mice were inoculated in the left rear footpad with 1,000 PFU of CHIKV. At days -1 and +2 relative to infection, mice were i.p. administered DT. (A) The percent starting body was determined daily. At 5 dpi, (B) viral RNA levels in the contralateral ankle were quantified by qRT-PCR and (C) infectious virus in the serum was quantified by plaque assay. Data are pooled from three independent experiments. *P* values were determined by a repeated measures two-way ANOVA with a Bonferroni’s multiple comparison test (A) and by Student’s t-test (B, C). *, *P* <0.05; **, *P* < 0.01; ***, *P* < 0.001.

Finally, to assess whether the depletion of CCR2^+^ cells impacts the control of other alphavirus infections, we infected DT-treated CCR2-DTR^+^ mice and their WT littermates with CHIKV. At 5 dpi, CHIKV-infected CCR2-DTR mice displayed impaired weight gain (Fig 5A) and increased viral burdens in the ankle (3-fold; *P* < 0.05) (Fig 5B) and the serum (*P* < 0.01) (Fig 5C) compared with CHIKV-infected WT mice, suggesting that Ly6C^hi^ inflammatory monocytes also contribute to the control of acute CHIKV infection.

### RRV-infected cells induce expression of type I IFN in monocytes by a MAVS-dependent mechanism

**Fig 6:**
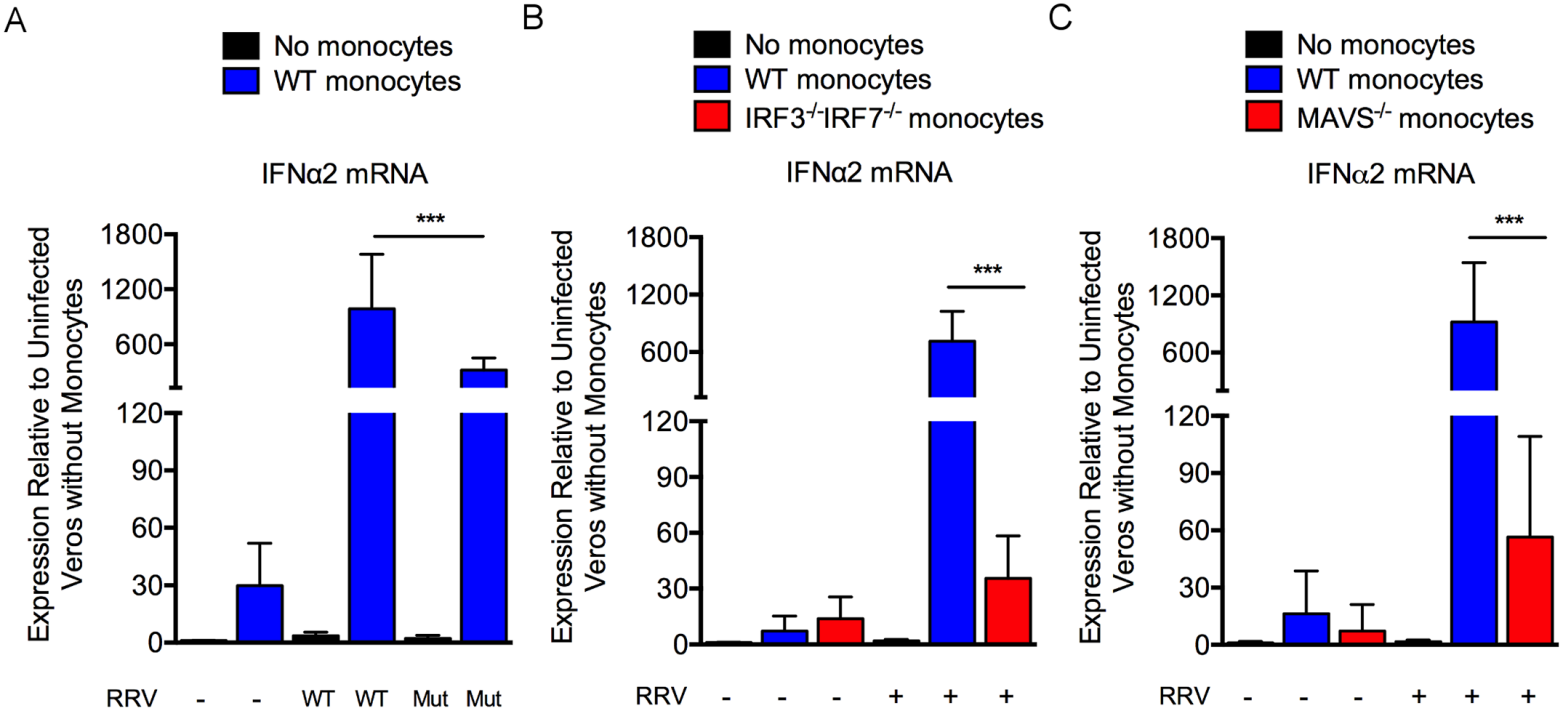
MAVS-dependent induction of type I IFN in monocytes during RRV infection. (A) IFNα2 mRNA expression levels in enriched WT bone marrow monocytes co-cultured for 18 h with uninfected, RRV-T48- or RRV-T48-nsP1^6M^-infected-infected Vero cells (n = 6/group). (B) IFNα2 mRNA expression levels in enriched WT or *Irf3*^-/-^;*Irf7*^-/-^ bone marrow monocytes co-cultured for 18 h with uninfected or RRV-T48-infected-infected Vero cells (n = 6/group). (C) IFNα2 mRNA expression levels in enriched WT or *Mavs*^-/-^ bone marrow monocytes co-cultured for 18 h with uninfected or RRV-T48-infected-infected Vero cells (n = 6-9/group). Data are normalized to 18S rRNA levels and are expressed as the relative expression (*n*-fold increase) over expression in uninfected Vero cells without monocytes. Data are combined from at least two independent experiments. *P* values were determined by one-way ANOVA with a Tukey’s multiple comparison test. *, *P* <0.05; **, *P* < 0.01; ***, *P* < 0.001.

In previous studies [54], we found that viral loads in tissues of RRV-T48- and RRV-T48-nsP1^6M^-infected *Ifnar1*^-/-^ mice, which lack the capacity to respond to type I IFNs [65], were equivalent, suggesting that the reduced viral loads of the nsP1 mutant virus in skeletal muscle tissue of WT mice are due to some aspect of the type I IFN response. To determine if monocytes are a source of type I IFN during RRV infection, we enriched mouse bone marrow monocytes and co-cultured them with RRV-infected Vero cells to mimic the events *in vivo* during which monocytes infiltrate RRV-infected tissues [16, 20, 28]. After 18 hours of co-culture, total cellular RNA was analyzed by qRT-PCR for expression of murine type I IFN. Importantly, given that Vero cells are primate in origin and lack the complete type I IFN gene cluster due to a homozygous deletion on chromosome 12 [66], the monocytes are the only source of murine interferons in the culture. We found that the expression level of IFNα2 mRNA was increased in WT monocytes co-cultured with RRV-T48- or RRV-T48-nsP1^6M^-infected Vero cells compared with monocytes co-cultured with uninfected Vero cells (Fig 6A), indicating that both viruses can elicit a type I IFN response in monocytes. We next investigated the signaling pathway leading to type I IFN gene expression in monocytes. As shown in Fig 6B, the induction of IFNα2 mRNA in monocytes was dependent on the transcription factors IRF3 and IRF7, as IFNα2 mRNA expression levels were reduced in *Irf3*^-/-^;*Irf7*^-/-^ monocytes compared with WT monocytes co-cultured with RRV-infected Vero cells. Similarly, the induction of IFNα2 mRNA was abrogated in *Mavs*^-/-^ monocytes co-cultured with RRV-infected Vero cells (Fig 6C). The reduced expression levels of IFNα2 mRNA in *Irf3*^-/-^;*Irf7*^-/-^ and *Mavs*^-/-^ monocytes was not due to a decrease in viability of these cells in the culture compared with WT monocytes (S8 Fig). These data suggest that the induction of type I IFN in monocytes in response to RRV is dependent on RIG-I like receptor (RLR) signaling through the adaptor molecule MAVS, resulting in IRF3 and/or IRF7-driven transcription of type I IFN genes.

### Induction of type I IFN in monocytes is dependent on infectious virus production

**Fig 7:**
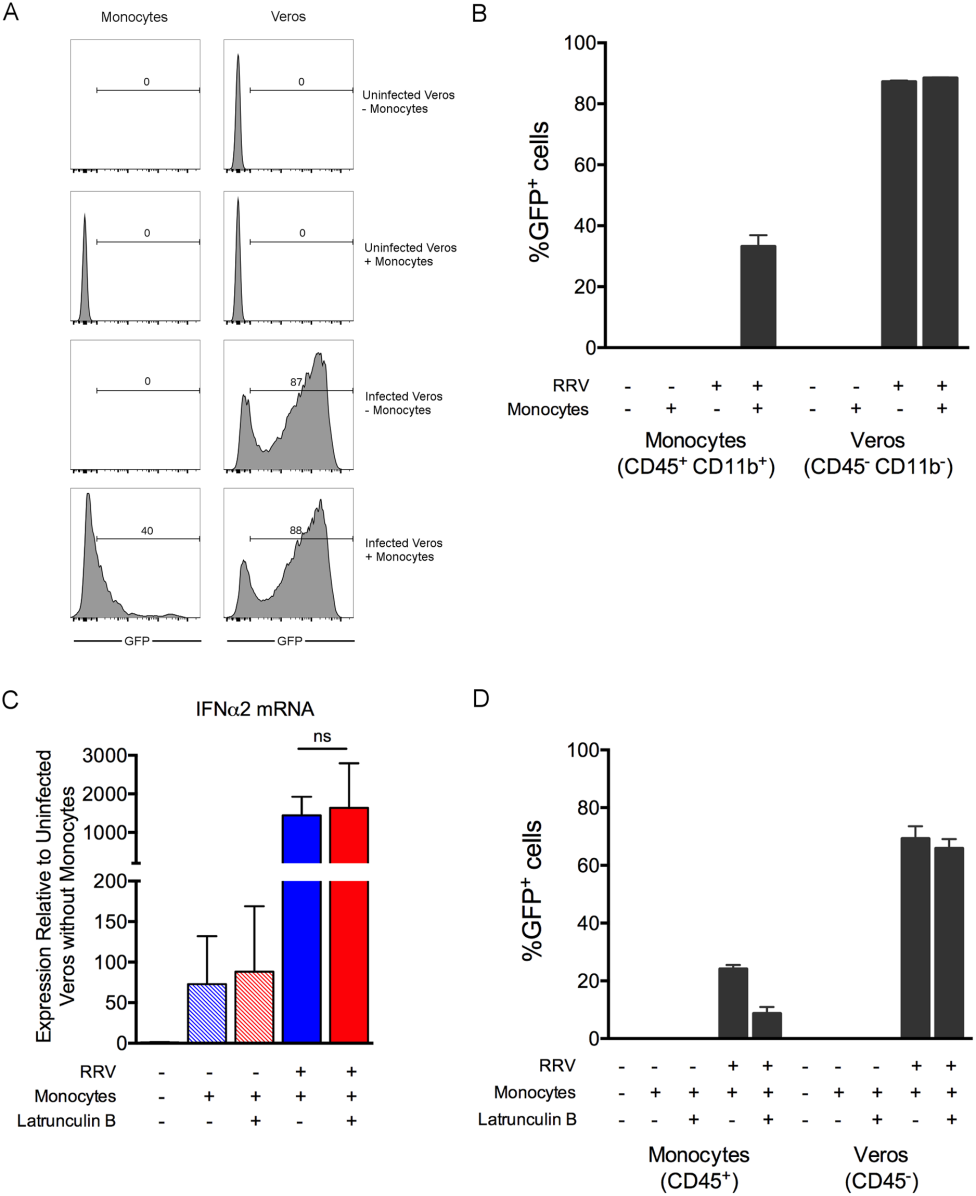
Induction of type I IFN expression in monocytes requires virus infection. (A-B) Enriched WT bone marrow monocytes were co-cultured with Vero cells infected with RRV-GFP (n = 3/group). After 18 h of co-culture, GFP expression in Vero cells and monocytes was measured by flow cytometry. (A) Shown are representative histograms. (B) The percent GFP^+^ monocytes and Vero cells. Data are representative of two independent experiments. (C-D) Enriched WT bone marrow monocytes were co-cultured with uninfected or RRV-GFP-infected Vero cells in the presence or absence of 1 μM Latrunculin B. After 18 h of co-culture, (C) IFNα2 mRNA expression level in monocytes was quantified by qRT-PCR. Data are normalized to 18S rRNA levels and are expressed as the relative expression (*n*-fold increase) over expression in uninfected Vero cells without monocytes. (D) The percent GFP^+^ Vero cells and monocytes were measured by flow cytometry based on GFP expression within the CD45^-^ Vero cells and the CD45^+^ monocytes. Data are combined from two independent experiments. *P* values were determined by one-way ANOVA with a Tukey’s multiple comparison test.

The requirement for *Mavs*, a component of the cytoplasmic viral RNA sensing pathway [67], in monocytes for the induction of IFNα2 mRNA in response to RRV infection suggested that direct viral infection of monocytes may be necessary for the induction of type I IFN. To assess whether the monocytes in this system are infected with RRV, we co-cultured monocytes with Vero cells infected with a RRV capable of expressing GFP under the control of a second subgenomic promoter (RRV-GFP) [28]. A portion of monocytes (~40%) co-cultured with Vero cells infected with RRV-GFP became GFP-positive, as measured by flow cytometry, although the fluorescence intensity of the GFP signal for many of the monocytes was reduced in comparison with RRV-GFP-infected Vero cells (Fig 7A and 7B). Although it remains possible that the monocytes phagocytosed GFP-positive Vero cells or engulfed GFP released from dying Vero cells, these data suggest that monocytes can be infected with RRV.

To further interrogate a role for direct infection, we first determined whether the induction of type I IFN in monocytes in the co-culture required the phagocytic activity of monocytes. Monocytes were co-cultured with Vero cells infected with RRV-GFP either in the presence or absence of the phagocytosis inhibitor Latrunculin B [68]. IFNα2 mRNA expression levels in monocytes were similar irrespective of the presence of Latrunculin B in the co-culture (Fig 7C). Latrunculin B treatment did result in a modest decrease in the percent of GFP^+^ monocytes (Fig 7D), suggesting that phagocytic uptake does occur but does not contribute to the induction of type I IFN. To further evaluate the role of direct infection of monocytes in the induction of type I IFN, monocytes were cultured in the presence of Vero cells electroporated with either RRV-GFP RNA or with RRV-GFP replicon RNA, in which the structural genes required for new particle production are replaced with the GFP gene [69]. Electroporation of Vero cells with full-length RRV-GFP RNA or with RRV-GFP replicon RNA resulted in a similar percentage of GFP-positive Vero cells (S9A Fig). However, monocytes co-cultured with Vero cells electroporated with viral RNA, but not replicon RNA, expressed IFNα2 mRNA (S9B Fig). Collectively, these data suggest that the induction of type I IFN in monocytes co-cultured with RRV-infected cells requires the production of virus particles that infect monocytes and activate type I IFN expression by a MAVS- and IRF3/IRF7-dependent mechanism.

### Ly6C^hi^ inflammatory monocytes are viral RNA positive and express *Irf7* during RRV infection *in vivo*

**Fig 8:**
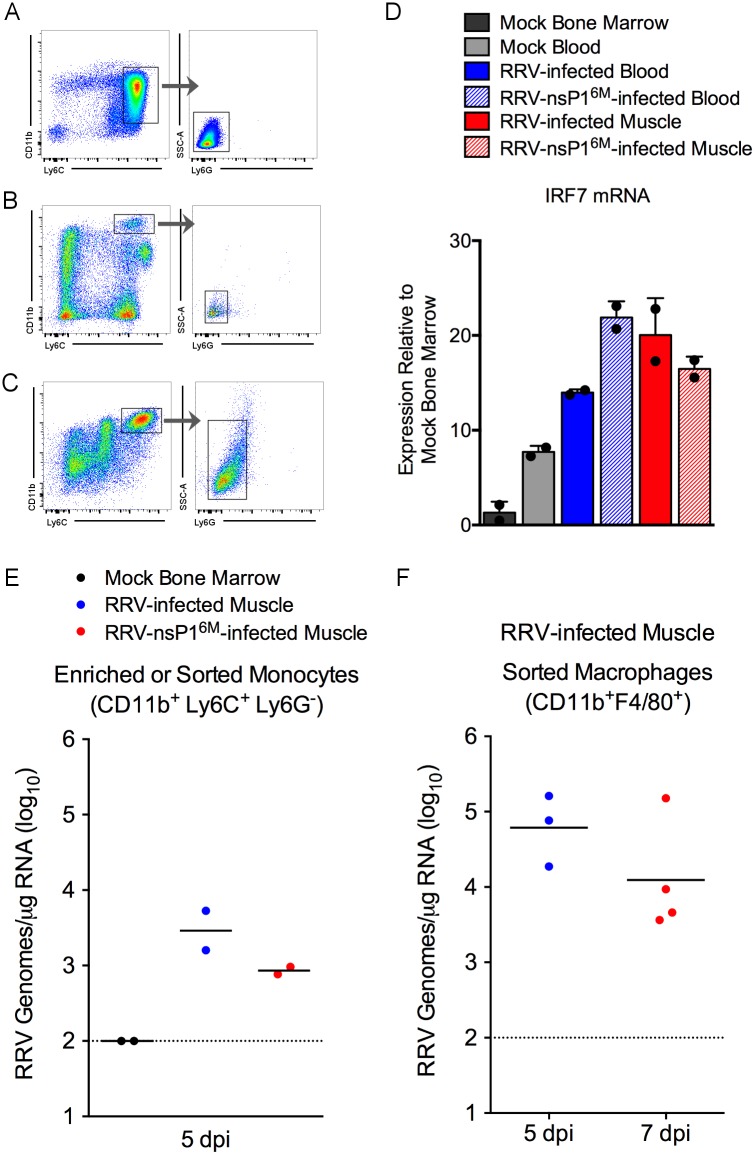
Inflammatory monocytes express *Irf7* and are viral RNA-positive during RRV infection *in vivo*. WT C57BL/6 mice were inoculated in the left rear footpad with PBS or 1,000 PFU of RRV-T48 or RRV-T48-nsP1^6M^. At 5 dpi, Ly6C^hi^ monocytes were enriched from the bone marrow or FACS-sorted from the blood and from enzymatically-digested muscle tissue. (A-C) Representative flow cytometry gating of cells sorted from the (A) bone marrow, (B) blood of RRV-infected mice, and (C) skeletal muscle of RRV-infected mice. (D) *Irf7* mRNA expression levels in enriched cell populations were quantified by qRT-PCR. Data are normalized to 18S rRNA levels and are expressed as the relative expression (*n*-fold increase) over expression in Ly6C^hi^ bone marrow monocytes from uninfected mice. Each data point represents monocytes sorted from the combined tissues of 3 mice. Each bar represents the arithmetic mean of two independent experiments. (E-F) RRV RNA levels in enriched monocyte/macrophage populations sorted from bone marrow of uninfected mice or skeletal muscle tissue of RRV-infected mice were quantified by qRT-PCR. (E) Each data point represents monocytes sorted from the combined muscle tissues of 3 mice. (F) Each data point represents monocytes/macrophages sorted from the muscle tissue of 2 mice (5 dpi) or a single mouse (7 dpi).

Given that Ly6C^hi^ monocytes express type I IFN in an IRF3/IRF7-dependent manner in response to co-culture with RRV-infected cells, we next assessed whether monocytes express *Irf7* and/or type I IFN during infection *in vivo*. WT C57BL/6 mice were mock-infected or infected with either RRV-T48 or RRV-T48-nsP1^6M^, and Ly6C^hi^ monocytes were enriched by FACS-sorting cells from the blood and the muscle tissue (RRV-infected mice only) at 5 dpi (Fig 8A–8C). As an additional control, Ly6C^hi^ monocytes were enriched from the bone marrow of mock-infected mice. In order to recover a sufficient number of monocytes via FACS sorting, tissues from 3 mice per group were combined into a single sample, and the experiment was performed twice. Total RNA was isolated from the sorted cells and analyzed by qRT-PCR for expression of murine type I IFN genes and *Irf7*. The expression of IFNβ, IFNα4, and IFNα2 were not detected in cells sorted from RRV-infected mice above levels detected in cells from mock-infected mice. Given that type I IFN mRNAs are tightly regulated [70] and that IRF7 has been reported to function as the main transcription factor driving type I IFN production during CHIKV infection [52], we analyzed expression of *Irf7* in the sorted cells. Ly6C^hi^ monocytes FACS-sorted from the blood and the muscle tissue of mice infected with either virus had higher levels of *Irf7* gene expression compared with monocytes from mock-infected mice (Fig 8D). These data suggest that *Irf7* is expressed in monocytes both in the circulation and at local sites of infection during RRV infection.

We next assessed whether the enriched Ly6C^hi^CD11b^+^Ly6G^-^ monocyte population was positive for viral RNA via qRT-PCR. Consistent with our studies *in vitro*, we found that monocytes sorted from skeletal muscle tissue of RRV-T48- or RRV-T48-nsP1^6M^-infected mice were positive for viral RNA (Fig 8E). To confirm these findings, we quantified RRV RNA in enriched CD11b^+^F4/80^+^ monocytes/macrophages that had been FACS-sorted from muscle tissue of RRV-T48-infected mice at either 5 or 7 dpi as previously described [71], and these cells also were positive for viral RNA (Fig 8F).

### MAVS deficiency results in increased viral burdens

**Fig 9:**
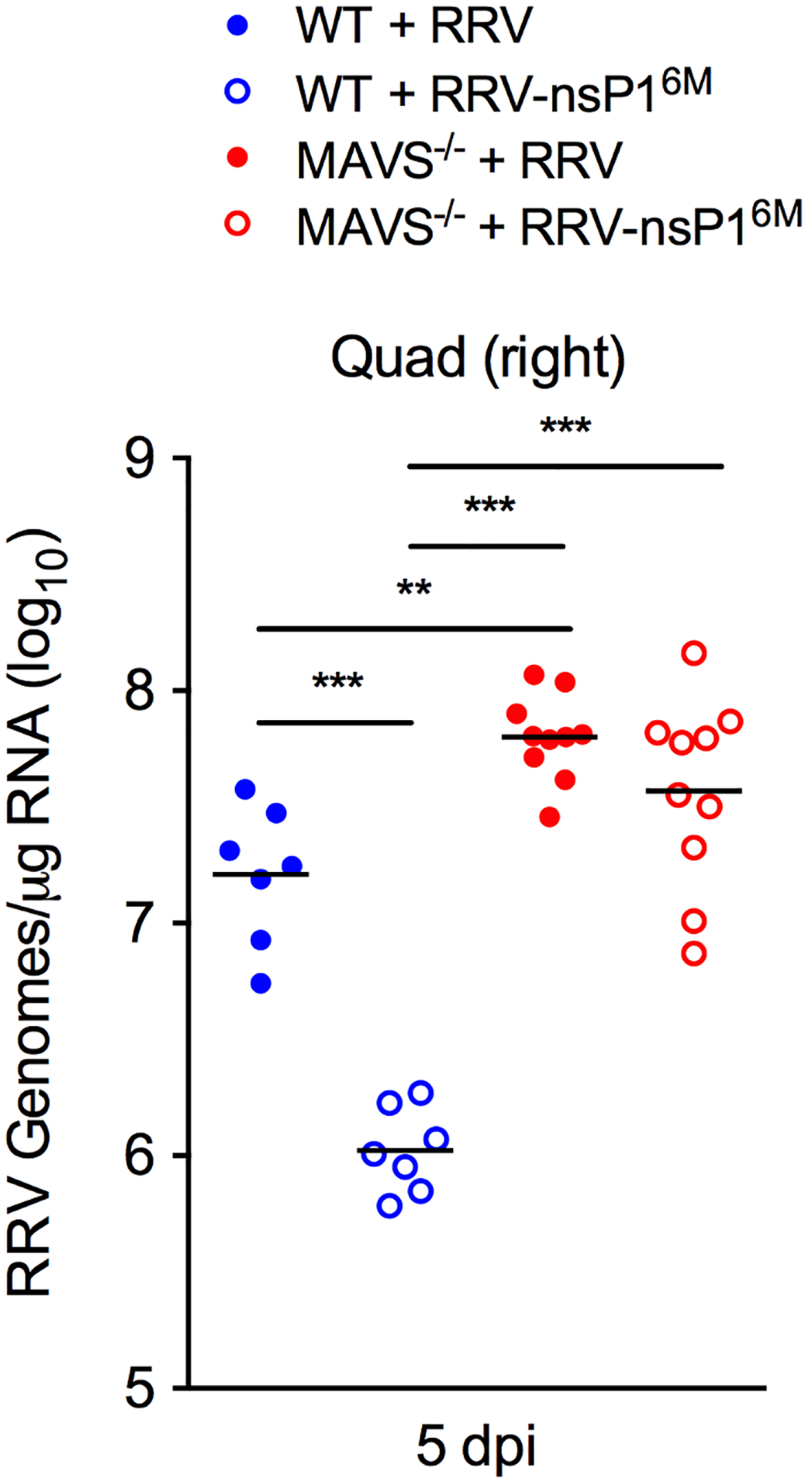
Viral loads of RRV-T48 and RRV-T48-nsP1^6M^ are equivalent in *Mavs*^-/-^ mice. WT (n = 7/group) or *Mavs*^-/-^ (n = 10/group) C57BL/6 mice were inoculated in the left rear footpad with 1,000 PFU of RRV-T48 or RRV-T48-nsP1^6M^. At 5 dpi, viral RNA levels in skeletal muscle tissue were quantified by qRT-PCR. Data are pooled from three independent experiments. *P* values were determined by one-way ANOVA with a Tukey’s multiple comparison test. **, *P* < 0.01; ***, *P* < 0.001.

Our experiments indicated that the nsP1 mutant virus is more sensitive to monocyte-mediated antiviral effects, that monocytes express type I IFN in response to RRV infection, and that the induction of type I IFN in monocytes during RRV infection occurs via a MAVS-dependent mechanism. Based on these data, we hypothesized that viral loads in tissues would be equivalent in RRV-T48 and RRV-T48-nsP1^6M^-infected *Mavs*^-/-^ mice. Accordingly, we infected WT and *Mavs*^-/-^ C57BL/6 mice with RRV-T48 or RRV-T48-nsP1^6M^ and measured viral burdens in skeletal muscle tissue at 5 dpi. Viral RNA levels in skeletal muscle tissue of RRV-T48-nsP1^6M^-infected WT mice at 5 dpi were reduced compared with RRV-T48-infected mice (15-fold; *P*, < 0.001) (Fig 9). Compared with viral RNA levels in RRV-T48-nsP1^6M^-infected WT mice, viral RNA levels in the skeletal muscle tissue of RRV-T48-nsP1^6M^-infected *Mavs*^-/-^ mice were increased (35-fold; *P*, < 0.001) (Fig 9) and were equivalent to viral RNA levels detected in the skeletal muscle tissue of RRV-T48-infected *Mavs*^-/-^ mice. These data suggest that the nsP1 mutant virus is more sensitive to MAVS-dependent antiviral effects and that determinants in nsP1 counteract MAVS-mediated viral restriction.

### CCR2^+^ hematopoietic cells protect *Irf3*^*-/-*^*;Irf7*^-/-^ mice from severe RRV infection

**Fig 10:**
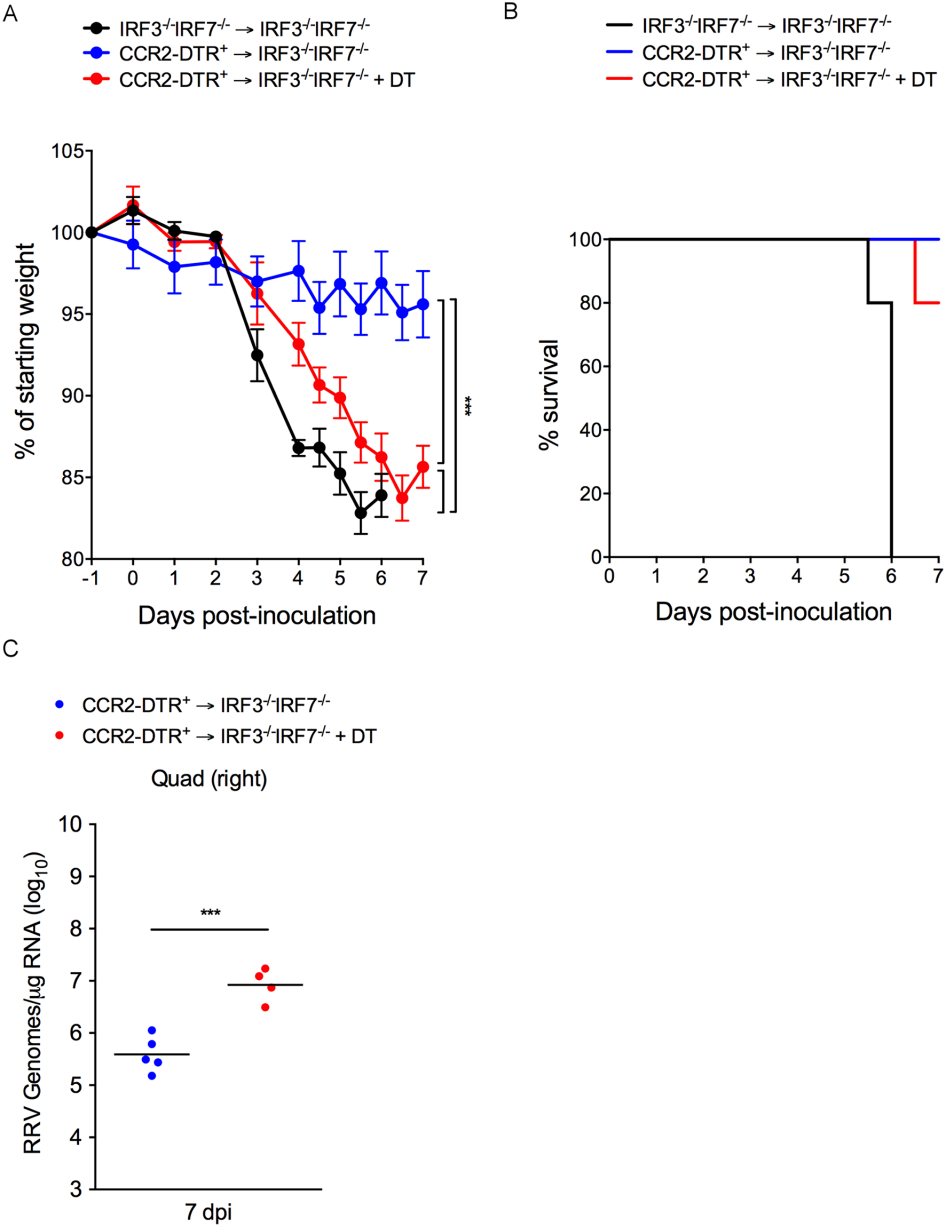
CCR2^+^ hematopoietic cells protect *Irf3*^*-/-*^;*Irf7*^-/-^ mice from severe RRV infection. *Irf3*^*-/-*^*;Irf7*^-/-^ C57BL/6 mice (n = 5/group) were lethally irradiated and reconstituted with *Irf3*^*-/-*^*;Irf7*^-/-^ or CCR2-DTR bone marrow. At 6 weeks post-irradiation, mice were inoculated in the left rear footpad with 10 PFU of RRV-T48-nsP1^6M^. At days -1 and +2 relative to infection, mice were i.p. administered PBS or DT. Mice were monitored daily for (A) morbidity and (B) mortality. *P* values were determined by two-way ANOVA with a Bonferroni’s multiple comparisons test. ***, *P* < 0.001. At 7 dpi, (C) viral RNA levels in skeletal muscle tissue were quantified by qRT-PCR and *P* values were determined by an unpaired t-test. ***, *P* < 0.001.

To further test the capacity of inflammatory monocytes to mount an antiviral response and protect against RRV infection *in vivo*, we generated *Irf3*^*-/-*^*;Irf7*^-/-^ → *Irf3*^*-/-*^*;Irf7*^-/-^ and CCR2-DTR → *Irf3*^*-/-*^*;Irf7*^-/-^ bone marrow chimeras. *Irf3*^*-/-*^*;Irf7*^-/-^ mice produce undetectable levels of type I IFN following alphavirus infection and are therefore highly susceptible to severe disease [51, 52]. Thus, this system tests the capacity of hematopoietic cells to protect from RRV infection in an IRF3/IRF7-dependent manner. To determine the percent chimerism in *Irf3*^*-/-*^*;Irf7*^-/-^ mice reconstituted with CCR2-DTR bone marrow, we took advantage of the fact that the CCR2-DTR allele also encodes CFP by using flow cytometry to analyze CFP expression in circulating monocytes (S10 Fig). At 5 weeks post-reconstitution, circulating monocytes were > 90% CFP-positive in all of the CCR2-DTR → *Irf3*^*-/-*^*;Irf7*^-/-^ bone marrow chimeras (S10 Fig). Bone marrow chimeric mice were i.p. administered PBS or DT one day prior and two days after inoculation with RRV-T48-nsP1^6M^. As expected, *Irf3*^*-/-*^*;Irf7*^-/-^ → *Irf3*^*-/-*^*;Irf7*^-/-^ mice developed severe disease and were humanely euthanized at 5–6 dpi (Fig 10A and 10B). In contrast, CCR2-DTR → *Irf3*^*-/-*^*;Irf7*^-/-^ mice treated with PBS were protected against severe RRV disease (Fig 10A and 10B), indicating that IRF3 and IRF7 sufficient hematopoietic cells can protect *Irf3*^*-/-*^*;Irf7*^-/-^ mice against RRV-T48-nsP1^6M^ infection. Treatment of CCR2-DTR → *Irf3*^*-/-*^*;Irf7*^-/-^ mice with DT resulted in more severe disease signs (Fig 10A and 10B) and higher viral loads in skeletal muscle tissue (Fig 10C) compared with CCR2-DTR → *Irf3*^*-/-*^*;Irf7*^-/-^ mice that did not receive DT. These data suggest that CCR2^+^ hematopoietic cells, including inflammatory monocytes, can protect from RRV infection via a type I IFN-dependent mechanism.

## Discussion

Infection with an arthritogenic alphavirus can result in acute and chronic pain and inflammation in musculoskeletal tissues. Numerous studies have demonstrated that arthritogenic alphaviruses target and replicate in musculoskeletal tissues, suggesting that the musculoskeletal disease is initiated by viral replication in the affected tissues. However, the immunological mechanisms that mediate control of alphavirus infection in musculoskeletal tissues are not well-defined. An improved understanding of these mechanisms may identify new therapeutic targets for the prevention or treatment of alphavirus-induced musculoskeletal disease.

In previous studies, we found that introduction of specific mutations in the nsP1 protein of the virulent RRV-T48 strain resulted in viral attenuation in mice, reduced viral loads in muscle tissue concurrent with immune cell infiltration, and increased viral sensitivity to type I IFN [54]. Thus, we reasoned that investigating the immunological mechanisms that mediate enhanced control of this attenuated virus would reveal aspects of the inflammatory response to RRV infection in musculoskeletal tissues that contribute to control of the infection. Indeed, we found that depletion of CCR2^+^ cells restored viral loads of the nsP1 mutant virus (RRV-T48-nsP1^6M^) in the muscle tissue at 5 days post-inoculation to levels detected in mice infected with WT RRV. At this time point, the immune cell infiltrate within musculoskeletal tissues is composed predominantly of monocytes, macrophages, and NK cells [20, 28]. Although both Ly6C^hi^ inflammatory monocytes and NK cells are effectively depleted in DT-treated CCR2-DTR mice, we have shown both previously [54] and in this study that antibody-mediated depletion of NK cells alone has no effect on viral loads or disease severity. In contrast, antibody-mediated depletion of monocytes and neutrophils, but not neutrophils alone, also increased viral loads and disease severity in mice infected with the nsP1 mutant virus. Collectively, these data suggest that the nsP1 mutant virus has enhanced sensitivity to the antiviral effects of Ly6C^hi^ monocytes.

Studies in mice using alternative methods to deplete or inhibit the migration of monocytes, as well as other myeloid cell populations, such as the administration of silica and carrageenan [27], clodronate-loaded lipsomes [19, 25], or the CCL2 inhibitor bindarit [46–48], suggested that monocytes or monocyte-derived cells are pathogenic effectors during arthritogenic alphavirus infections. However, the precise effects of these broadly-acting depletion methods on different immune cell subsets, including monocytes, dendritic cells, and macrophages, in the context of these infections has not been extensively investigated. Additionally, the mechanism(s) by which bindarit acts to inhibit chemokine production is not well-defined, although modulation of the classical NF-κB pathway has been reported [72], suggesting that other mechanisms in conjunction with CCL2 inhibition may contribute to the reduced disease severity observed in RRV- or CHIKV-infected mice treated with bindarit. In addition, CHIKV-induced musculoskeletal disease was more severe in *Ccr2*^-/-^ mice [14], which displayed diminished recruitment of monocytes and enhanced recruitment of neutrophils to tissues, suggesting that monocytes may protect from more severe forms of CHIKV-induced musculoskeletal disease. Collectively, these studies, combined with the findings reported in this study, suggest that monocytes may serve both pathogenic and protective roles during arthritogenic alphavirus infections. Indeed, monocytes have been implicated previously in contributing to both protection and pathology during viral infections of the CNS [73].

As discussed above, in previous studies we found that the nsP1 mutant virus was more sensitive to the antiviral effects of type I IFN *in vitro* and that viral loads of WT RRV and the nsP1 mutant virus in skeletal muscle tissue were similar in *Ifnar1*^-/-^ mice [54]. The restoration of viral loads of RRV-T48-nsP1^6M^ to WT RRV levels in both *Ifnar1*^-/-^ mice and mice depleted of inflammatory monocytes suggested that monocytes are a source of type I IFN. *In vitro*, we found that co-culture of Ly6C^hi^ monocytes with RRV-infected cells resulted in the induction of type I IFN gene expression in monocytes. These findings are consistent with studies showing that human monocytes produce type I IFNs in response to infection or exposure to CHIKV [56, 57]. The induction of type I IFN in Ly6C^hi^ monocytes was dependent on the transcription factors IRF3 and IRF7 and the signaling adaptor MAVS. These findings imply that the expression of type I IFN is triggered by viral infection of the monocytes, with subsequent sensing of viral RNA present in the cytoplasm by a RIG-I-like receptor (RLR) that signals via MAVS. Consistent with this model, human monocytes and monocyte-derived cell lines have been reported to be susceptible to infection by CHIKV and RRV [56, 57, 74]. However, whether the infection of human monocytes results in productive virus replication is less clear. Monocytes in PBMCs isolated from CHIKV-infected patients stained positive for CHIKV antigen by flow cytometry [56], and infection of the human myeloid MM6 cell line with RRV resulted in an infection in which viral RNA was detectable, but production of infectious virus was low [74], suggesting that productive viral replication in myeloid cells may be restricted compared to infection in other cell types. Using flow cytometry, we found that a subset of murine Ly6C^hi^ monocytes became GFP-positive when co-cultured with cells infected with a recombinant RRV that expressed GFP from a second subgenomic reporter. We also found that the induction of type I IFN in Ly6C^hi^ monocytes was dependent on viral particle production, as co-culture of Ly6C^hi^ monocytes with cells electroporated with full-length viral RNA, but not replicon RNA incapable of progeny virus production, resulted in the induction of type I IFN expression in the monocytes.

Monocytes as a source of type I IFN during alphavirus infection is supported further by several experiments *in vivo*. First, we found that Ly6C^hi^ monocytes sorted from both the blood and muscle tissue of mice infected with RRV expressed higher levels of *Irf7* mRNA, a critical regulator of type I IFN production in CHIKV-infected mice [52], compared with Ly6C^hi^ monocytes sorted from control mice. Second, similar to mice depleted of Ly6C^hi^ monocytes, we found that viral loads in the muscle tissue of *Mavs*^-/-^ mice infected with WT RRV or the nsP1 mutant virus were equivalent. Third, consistent with previous studies showing that WT bone marrow can rescue *Irf3*^*-/-*^;*Irf7*^-/-^ mice from lethal CHIKV infection [51], we found that lethally-irradiated *Irf3*^*-/-*^;*Irf7*^-/-^ mice reconstituted with CCR2-DTR bone marrow were protected from severe RRV infection. These studies suggesting that type I IFN production by hematopoietic cells is sufficient to control acute alphavirus infection. Finally, DT treatment of *Irf3*^*-/-*^;*Irf7*^-/-^ mice reconstituted with CCR2-DTR bone marrow strongly enhanced disease severity and viral loads in skeletal muscle tissue, suggesting that CCR2^+^ hematopoietic cells, including inflammatory monocytes, are capable of inducing an antiviral response. These findings, together with our findings that induction of type I IFN in monocytes exposed to RRV-infected cells is IRF3/IRF7 and MAVS-dependent, suggest an important role for MAVS-mediated type I IFN production by Ly6C^hi^ monocytes for control of acute alphavirus infection.

In summary, these studies have defined an important antiviral function for inflammatory Ly6C^hi^ monocytes in acute RRV and CHIKV infection, whereby monocytes mediate improved virus control and reduced viral disease severity. In the case of RRV, these studies also suggest that the antiviral activity of monocytes can be subverted by determinants in the nsP1 protein, which mediates capping of viral RNAs that potentially influence host recognition of infection. In combination with previous studies that suggested additional protective roles for inflammatory monocytes during CHIKV infection [14], our findings further indicate that the development of therapeutics targeting monocyte recruitment or function for the treatment of these infections should do so without compromising the roles of these cells in innate immunity.

## Materials and methods

### Ethics statement

This study was conducted in accordance with the recommendations in the Guide for the Care and Use of Laboratory Animals and the American Veterinary Medical Association (AVMA) Guidelines for the Euthanasia of Animals. All animal experiments were performed with the approval of the Institutional Animal Care and Use Committee at the University of Colorado School of Medicine (Assurance Number: A3269-01) under protocols B-86514(10)1E and B-86517(03)1E. Experimental animals were humanely euthanized at defined endpoints by exposure to isoflurane vapors followed by thoracotomy.

### Viruses

The T48 stain of RRV (GenBank accession no. GQ433359) was isolated from *Aedes vigilax* mosquitoes in Queensland, Australia [75]. Prior to cDNA cloning, the virus was passaged 10 times in suckling mice, followed by two passages on Vero cells [76, 77]. RRV strain DC5692 (GenBank accession no. HM234643), from which the attenuating mutations in nsP1 were derived [54, 58], was isolated in 1995 from *Aedes camptorhynchus* mosquitoes at Dawesville Cut in the Peel region of Western Australia [78]. The virus was passaged once in C6/36 cells, once in Vero cells (ATCC CCL-81), and once in BHK-21 [C-13] (ATCC CCL-10) cells prior to cDNA cloning [58]. RRV-T48-nsP1^6M^ encodes the six nonsynonymous DC5692 nucleotides in the nsP1 gene [54]. The SL15649 strain of CHIKV (GenBank accession no. GU189061) was isolated from a serum sample collected from a febrile patient in Sri Lanka in 2006. This virus was passaged two times in Vero cells prior to cDNA cloning [26]. Plasmids encoding infectious cDNA clones of RRV and CHIKV have been described [26, 54, 58, 76]. Virus stocks were prepared from cDNA clones as previously described [26, 28]. Briefly, plasmids were linearized and used as a template for *in vitro* transcription with SP6 DNA-dependent RNA polymerase (Ambion). RNA was electroporated into BHK-21 cells and at 24 h post-electroporation, cell culture supernatant was collected and clarified by centrifugation at 1,721 x *g*. Clarified supernatants were aliquoted and stored at -80°C. Viral titers were determined by plaque assays using BHK-21 and Vero cells as described [26, 64].

### Mouse experiments

Mice were bred in specific-pathogen-free facilities at the University of Colorado Anschutz Medical Campus. All mouse studies were performed in an animal biosafety level 2 or 3 laboratory. WT and *Rag1*^-/-^ C57BL/6J mice were obtained from the Jackson Laboratory. CCR2-DTR mice, in which one CCR2 locus was modified to encode a simian DT receptor (DTR) and enhanced cyan fluorescent protein [59], were provided by Eric G. Pamer (Memorial Sloan-Kettering Cancer Center). *Mavs*^-/-^ C57BL/6 mice, originally generated at the University of Washington, were provided by Kenneth Tyler (University of Colorado School of Medicine). *Irf3*^-/-^;*Irf7*^-/-^ C57BL/6 mice were provided by Michael S. Diamond (Washington University). Three-to-four week-old mice were used for all studies. Mice were inoculated in the left rear footpad with 10^3^ PFU of virus in diluent (PBS supplemented with 1% FBS). Mock-infected animals received diluent alone. Disease scores of RRV-infected mice were determined by assessing grip strength, hind limb weakness, and altered gait, as previously described [58]. Briefly, grip strength and hind limb weakness were assessed by testing the ability of each mouse to grip a rounded surface. Mice were scored as follows: 0, no disease signs; 1, extremely mild defect in injected hind paw gripping ability; 2, very mild defect in bilateral hind paw gripping ability; 3, bilateral loss of gripping ability and mild bilateral hind limb paresis; 4, bilateral loss of gripping ability, moderate bilateral hind limb paresis, observable altered gait, and difficulty or failure to right self; 5, bilateral loss of gripping ability, severe bilateral hind limb paresis, altered gait with hind paw dragging, and failure to right self; 6, bilateral loss of gripping ability, very severe hind limb paresis with dragging of limbs, and failure to right self. On the day of experiment termination, mice were sedated with isoflurane and euthanized by thoracotomy, blood was collected, and mice were perfused by intracardiac injection of 1x PBS. PBS-perfused tissues were removed by dissection and homogenized in TRIzol Reagent (Life Technologies) for RNA isolation using a MagNA Lyser (Roche). For cell depletion experiments in CCR2-DTR mice, mice were injected i.p. with 10 ng/g body weight diphtheria toxin (DT) per injection. For antibody depletion experiments, mice were injected with 300 μg/mouse of either anti-NK1.1 (clone PK136, BioXcell), anti-Gr1 (clone RB6-8C5, BioXcell), anti-Ly6G (clone 1A8, BioXcell), or similar amounts of appropriate control antibody per injection.

### Generation of bone marrow chimeras

Eight-ten week old *Irf3*^-/-^;*Irf7*^-/-^ C57BL/6 recipient mice received 1,000 rad (2 doses of 500 rad each) prior to i.v. reconstitution with 6 x 10^6^ *Irf3*^-/-^;*Irf7*^-/-^ or CCR2-DTR bone marrow cells depleted of CD90.2^+^ cells using anti-CD90.2-conjugated magnetic beads (Miltenyi Biotec). Five weeks after reconstitution, successful chimerism in the mice receiving CCR2-DTR bone marrow was assessed by measuring CFP expression, which is expressed by the CCR2-DTR allele, in circulating CD11b^+^Ly6C^hi^ monocytes by flow cytometry (S10 Fig). Six weeks post-reconstitution, bone marrow chimeras were inoculated with 10 PFU of RRV-nsP1^6M^ in the left rear footpad. Mice were i.p. inoculated with 1x PBS or 10 ng/g body weight DT at day -1 and day +2 relative to virus inoculation.

### Enrichment of monocytes and adoptive transfer

CCR2-DTR mice were treated with DT as described above. Monocytes were enriched from the bone marrow of WT C57BL/6 mice using a negative selection monocyte isolation kit (Miltenyi Biotec). The purity of negatively-selected monocytes was assessed by flow cytometry and defined as %CD11b^+^Ly6C^hi^Ly6G^-^ of total singlets (S5 Fig). 2 x 10^6^ enriched monocytes per mouse were adoptively transferred into a subset of CCR2-DTR mice immediately prior to inoculation with virus.

### Quantification of viral RNA

RNA was isolated from TRIzol homogenized tissues using a PureLink RNA Mini Kit (Life Technologies). Absolute quantification of RRV RNA was performed as previously described [71]. A sequence-tagged (small caps) RRV-specific RT primer (4415 5’-ggcagtatcgtgaattcgatgcAACACTCCCGTCGACAACAGA-3’) was used for reverse transcription. A tag sequence-specific reverse primer (5’-GGCAGTATCGTGAATTCGATGC-3’) was used with a RRV sequence-specific forward primer (4346 5’-CCGTGGCGGGTATTATCAAT-3’) and an internal TaqMan probe (4375 5’-ATTAAGAGTGTAGCCATCC-3’) during qPCR to enhance specificity. To create standard curves, 10-fold dilutions, from 10^8^ to 10^2^ copies, of RRV genomic RNAs synthesized *in vitro* were spiked into RNA from BHK-21 cells and reverse transcription and qPCR were performed in an identical manner. The limit of detection was 100 genome copies. CHIKV RNA in tissues was quantified by qRT-PCR as previously described [79].

### Plaque assays

Virus-containing samples were serially diluted 10-fold, and dilutions were adsorbed onto BHK-21 monolayers for 1 h at 37°C. Monolayers were then overlayed with medium containing 0.5% immunodiffusion agarose and incubated at 37°C for 36–40 hours. Plaques were visualized using Neutral Red staining and enumerated to determine the PFU/ml of each sample.

### Flow cytometry

Quadriceps muscles were dissected, minced, and incubated for 1.5 h with vigorous shaking at 37°C in digestion media (RPMI 1640, 10% FBS, 15 mM HEPES, 2.5 mg/ml Collagenase Type 1 [Worthington Biochemical], 1.7 mg/ml DNase I [Roche], 1x gentamicin [Life Technologies], 1% penicillin/streptomycin). Following digestion, cells were passed through a 100-μm cell strainer (BD Falcon) and banded on Lympholyte-M (Cedarlane Laboratories) to isolate infiltrating leukocytes. Additionally, peripheral blood was collected at time of harvest in 100 mM EDTA, and red blood cells were lysed using BD PharmLyse buffer (BD Biosciences). Spleens were dissected from mice and passed through a 100-μm cell strainer. Following red blood cell lysis, cells were washed in 1x PBS and passed through a 70-μm strainer, and total viable cells were determined by trypan blue exclusion. Isolated leukocytes from all samples were incubated with anti-mouse FcγRII/III (2.4G2; BD Pharmingen) for 20 min on ice to block nonspecific antibody binding and then stained in FACS buffer (1x PBS, 2% FBS) with the following Abs: anti-B220 (RA3-6B2), anti-CD11b (M1/70), anti-F4/80 (BM8), anti-Ly6C (HK1.4), anti-Ly6G (1A8), anti-CD43 (1B11), anti-CD64 (X54-5/7.1), anti-CD11c (N418), anti-NK1.1 (PK136), anti-CD45 (30-F11), anti-CD45.1 (A20), and anti-CD45.2 (104). Cells were fixed overnight in 1% paraformaldehyde and analyzed on an LSRFortessa using FACSDiva software (Becton Dickinson). Further analysis was done using FlowJo Software (Tree Star).

### FACS sorting

Muscle and peripheral blood samples were obtained and processed as described above. Total viable cells were determined by trypan blue exclusion. Isolated leukocytes from all samples were incubated with LIVE/DEAD Violet Dead Cell Stain (Molecular Probes) for 30 min on ice in 1x PBS to identify dead cells. Isolated leukocytes were incubated with anti-mouse FcγRII/III (2.4G2; BD Pharmingen) for 20 min on ice to block nonspecific Ab binding and then stained in FACS buffer (1x PBS, 2% FBS) with the following Abs: anti-CD11b (M1/70), anti-Ly6C (HK1.4), and anti-Ly6G (1A8). Cells were then analyzed and sorted on a FACSAria sorter using FACSDiva software (Becton Dickinson).

### Monocyte isolation and co-culture

Vero cells were plated in 12-well plates at a density of 2 x 10^5^ cells per well the day prior to infection (MOI of 1 PFU/cell). In some experiments, Vero cells were electroporated with viral RNA, then plated at a density of 3 x 10^5^ cells per well 5 hours prior to the addition of enriched monocytes. Monocytes were isolated from mouse bone marrow using the EasySep Mouse Monocyte Isolation Kit (Stem Cell Technologies) according to the manufacturer’s instructions. The degree of monocyte enrichment was assessed by flow cytometry, and the average purity of the enriched population was 91% Ly6C^hi^ monocytes. Enriched bone marrow monocytes were plated with Vero cells at a density of 1 x 10^5^ monocytes per well 5 hours after viral infection of the Vero cells. In some experiments, monocytes were added to the Vero cells in the presence of 1 uM Latrunculin B (Sigma). Co-cultures were harvested 18 hours after plating of the monocytes for analysis. Cells were harvested either into TRIzol reagent for RNA isolation and qRT-PCR analysis or collected for staining and flow cytometry analysis.

### qRT-PCR (Host genes)

RNA was isolated from cells collected in TRIzol reagent using the PureLink RNA Mini Kit (Life Technologies). Random primers (Life Technologies) were used to generate random-primed cDNAs, which were then subjected to qPCR analysis using TaqMan primer/probe sets specific to 18S rRNA, murine IFNα2, IFNβ, IFNα4, or Irf7 (Applied Biosystems). IFNα2, IFNβ, IFNα4, or Irf7 expression was normalized to 18S values to control for input amounts of cDNA. The relative fold induction of amplified mRNA were determined by using the Ct method [80].

### Statistical analysis

All data were analyzed using GraphPad Prism 6 software. Data were evaluated for statistically significant differences using a two-tailed, unpaired t test, a one-way analysis of variance (ANOVA) test followed by Tukey’s multiple comparison test, or a two-way ANOVA followed by a Bonferroni multiple comparison test. A *P*-value < 0.05 was considered statistically significant. All differences not specifically indicated to be significant were not significant (P > 0.05).

## Supporting information

S1 Fig Flow cytometry gating of monocytes, neutrophils, and NK cells in blood. WT (n = 6-7/group) or CCR2-DTR (n = 5-6/group) C57BL/6 mice were inoculated in the left rear footpad with (A) PBS, (B) RRV-T48, or (C) CHIKV. At days -1 and +2 relative to infection, mice were i.p. administered DT. At 24 h after the last DT administration, the frequency of Ly6C^hi^ monocytes (Ly6C^hi^CD11b^+^CD43^+^Ly6G^-^), NK cells (NK1.1^+^CD11b^+^Ly6C^-^Ly6G^-^), and neutrophils (Ly6G^+^CD11b^+^CD43^+^Ly6C^+^) in the blood were determined by the flow cytometry gating shown.
(TIF)

S2 Fig Ly6C^hi^ monocytes and NK cells in CCR2-DTR mice express CFP. CFP expression in (A) Ly6C^hi^ monocytes (Ly6C^hi^CD11b^+^CD43^+^Ly6G^-^), (B) NK cells (NK1.1^+^CD11b^+^Ly6C^-^Ly6G^-^), and (C) neutrophils (Ly6G^+^CD11b^+^CD43^+^Ly6C^+^) in the blood of WT (CCR2-DTR-CFP^-^) (n = 3) and CCR2-DTR-CFP^+^ (n = 5) C57BL/6 mice was determined by flow cytometry.
(TIF)

S3 Fig Flow cytometry gating of leukocytes in skeletal muscle tissue. WT (n = 4–5 mice/group) or CCR2-DTR (n = 2–5 mice/group) C57BL/6 mice were inoculated in the left rear footpad with either PBS or RRV-T48. DT was administered at day -1 and day +2 post-inoculation. At 48 h after the last DT administration, the number of NK cells (NK1.1^+^CD11b^+^Ly6C^-^Ly6G^-^), neutrophils (Ly6G^+^CD11b^+^CD43^+^Ly6C^+^), and various Ly6C^+^CD11b^+^ and Ly6C^hi^CD11b^+^ myeloid subsets were determined by flow cytometry using the gating strategy shown.
(TIF)

S4 Fig Weight gain of control and DT-treated CCR2-DTR mice. WT (n = 7) or CCR2-DTR^+^ (n = 10) C57BL/6 mice were inoculated in the left rear footpad with PBS. At days -1 and +2 relative to PBS inoculation, mice were i.p. administered DT. The percent starting body was determined daily. Data are pooled from three independent experiments.
(TIF)

S5 Fig Enriched Ly6C^hi^ monocytes for adoptive transfer. The purity of Ly6C^hi^ monocytes isolated from the bone marrow was assessed by flow cytometry. Cells were incubated with anti-mouse FcγRII/III to block nonspecific antibody binding and then stained with the following antibodies: anti-CD11b (M1/70), anti-Ly6C (HK1.4), and anti-Ly6G (1A8). Shown are representative FACS plots from one of three independent experiments.
(TIF)

S6 Fig Inflammatory monocytes control RRV infection in *Rag1*^-/-^ mice. *Rag1*^-/-^ (n = 6-8/group) or *Rag1*^-/-^;CCR2-DTR^+^ (n = 5-6/group) C57BL/6 mice were inoculated in the left rear footpad with 1,000 PFU of RRV-T48 or RRV-T48-nsP1^6M^. At days -1 and +2 relative to infection, mice were i.p. administered DT. (A) At 5 dpi, viral RNA levels in skeletal muscle tissue were quantified by qRT-PCR. (B) The percent starting body weight was determined daily. Data are pooled from two independent experiments. Statistical comparisons shown are between RRV-T48 in *Rag1*^-/-^ mice versus *Rag1*^-/-^;CCR2-DTR mice (black) and between RRV-T48-nsP1^6M^ in *Rag1*^-/-^ mice versus *Rag1*^-/-^;CCR2-DTR mice (gray). *P* values were determined by one-way ANOVA with a Tukey’s multiple comparison test (A) and a repeated measures two-way ANOVA with a Bonferroni’s multiple comparison test (B).). *, *P* < 0.05; ***, *P* < 0.001.
(TIF)

S7 Fig Antibody-mediated depletion of NK cells, monocytes, and neutrophils. WT C57BL/6 mice were administered (A) anti-NK1.1 (n = 4) or a control antibody (n = 4), (B) anti-Gr1 (n = 8) or a control antibody (n = 8), or (C) anti-Ly6G (n = 4) or a control antibody (n = 4) at day -1 and day +2 relative to infection with the indicated viruses. At 5 dpi, depletion of NK cells, neutrophils, and Ly6C^hi^ monocytes in the circulation was assessed by flow cytometry.
(TIF)

S8 Fig Monocyte viability in co-culture assays. Bone marrow monocytes from WT, *Irf3*^-/-^;*Irf7*^-/-^, and *Mavs*^-/-^ mice were co-cultured for 18 h with uninfected or RRV-T48-infected Vero cells. Cells were incubated with LIVE/DEAD Violet Dead Cell Stain, incubated with anti-mouse FcγRII/III to block nonspecific antibody binding, and then stained with the following antibodies: anti-CD11b (M1/70), anti-Ly6C (HK1.4), and anti-CD45 (30-F11). The percent viability of Ly6C^hi^CD11b^+^CD45^+^ cells was determined by flow cytometry (n = 3/group).
(TIF)

S9 Fig Induction of type I IFN expression in monocytes co-cultured with RRV-infected Vero cells requires new virion production. (A-B) Enriched WT bone marrow monocytes were co-cultured with Vero cells electroporated with full-length RRV-GFP RNA or replicon RRV-GFP RNA in which the structural genes are replaced with the GFP gene. After 18 h of co-culture, (A) the percent GFP^+^ Vero cells was measured by flow cytometry, and (B) IFNα2 mRNA expression level in monocytes was quantified by qRT-PCR. Data are normalized to 18S rRNA levels and are expressed as the relative expression (*n*-fold increase) over expression in uninfected Vero cells without monocytes. *P* values were determined by one-way ANOVA with a Tukey’s multiple comparison test. ***, *P* < 0.001.
(TIF)

S10 Fig Chimerism of *Irf3*^-/-^;*Irf7*^-/-^ mice reconstituted with CCR2-DTR bone marrow. (A-B) Five weeks after reconstitution, successful chimerism in mice receiving CCR2-DTR bone marrow was assessed by measuring CFP expression in circulating CD11b^+^Ly6C^hi^ monocytes by flow cytometry. (A) The gating scheme used to define CFP expression in Ly6C^hi^ monocytes. (B) Percent CFP expression within the Ly6C^hi^ monocyte gate shown in (A).
(TIF)
