# Supplementary figures and images for: Inflammatory monocytes mediate control of acute alphavirus infection in mice
Source: PLoS Pathog. 2017 Dec 15;13(12):e1006748. doi: 10.1371/journal.ppat.1006748 (PMC5747464; doi:10.1371/journal.ppat.1006748)

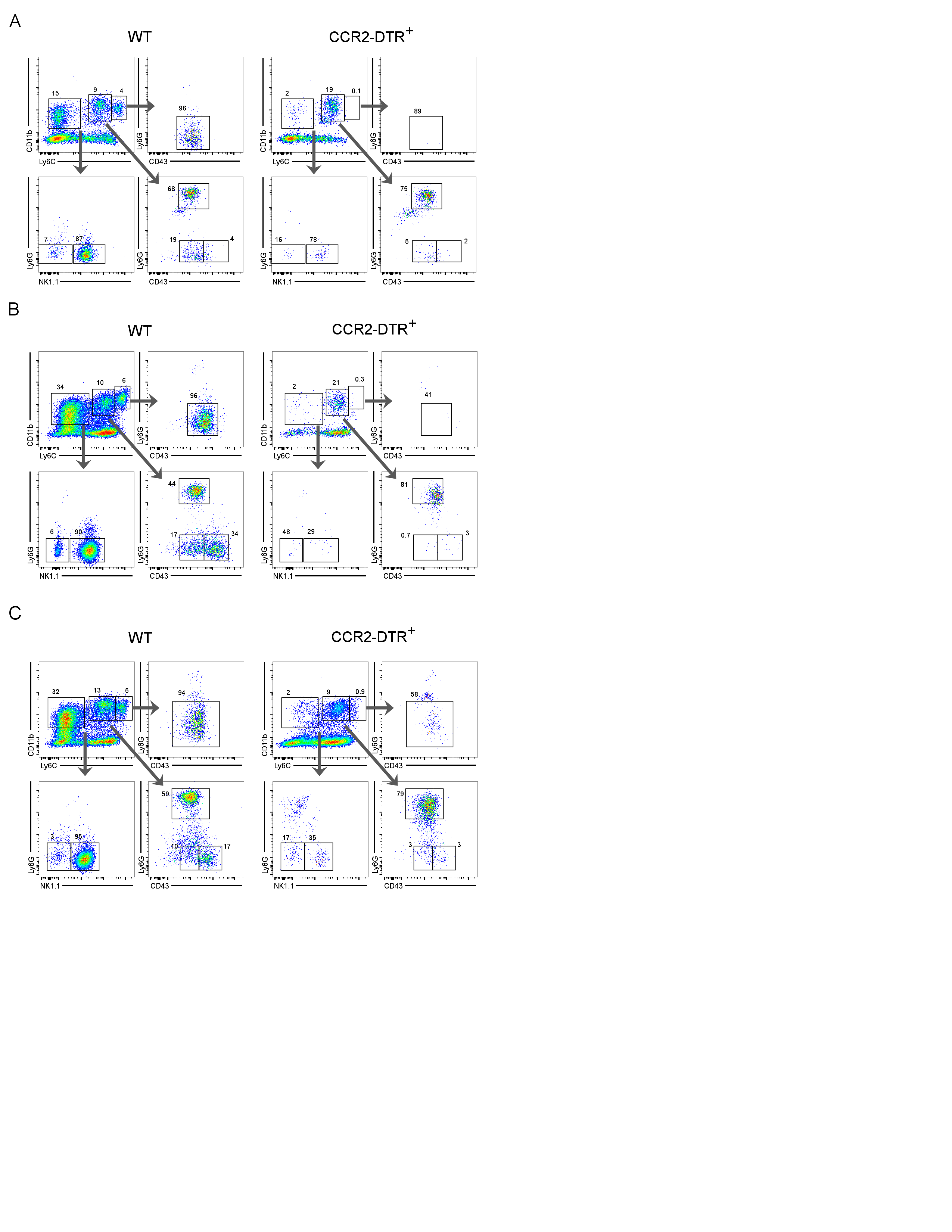

Supplement: S1 Fig — WT (n = 6-7/group) or CCR2-DTR (n = 5-6/group) C57BL/6 mice were inoculated in the left rear footpad with (A) PBS, (B) RRV-T48, or (C) CHIKV. At days -1 and +2 relative to infection, mice were i.p. administered DT. At 24 h after the last DT administration, the frequency of Ly6Chi monocytes (Ly6ChiCD11b+CD43+Ly6G-), NK cells (NK1.1+CD11b+Ly6C-Ly6G-), and neutrophils (Ly6G+CD11b+CD43+Ly6C+) in the blood were determined by the flow cytometry gating shown. (TIF) [file ppat.1006748.s001.tif]

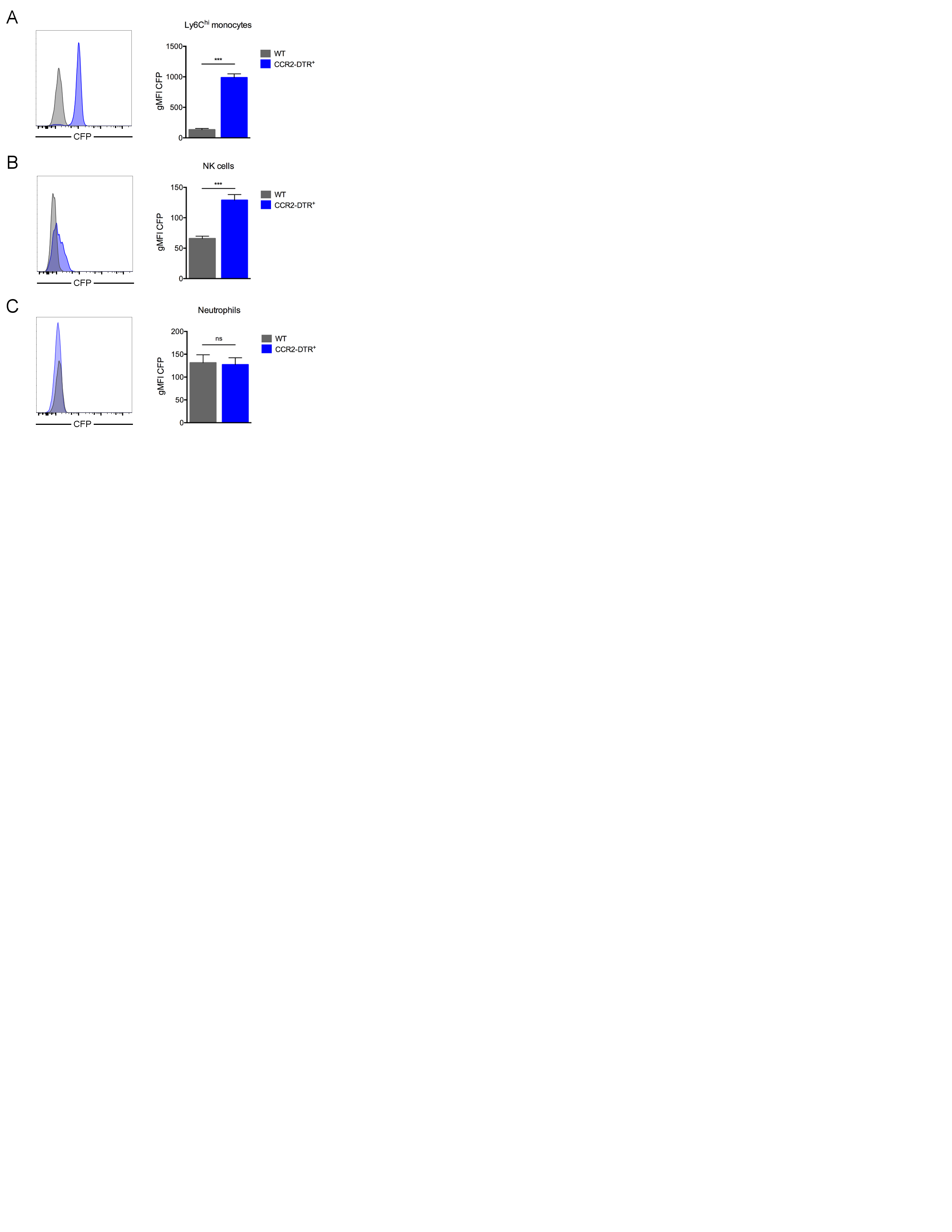

Supplement: S2 Fig — CFP expression in (A) Ly6Chi monocytes (Ly6ChiCD11b+CD43+Ly6G-), (B) NK cells (NK1.1+CD11b+Ly6C-Ly6G-), and (C) neutrophils (Ly6G+CD11b+CD43+Ly6C+) in the blood of WT (CCR2-DTR-CFP-) (n = 3) and CCR2-DTR-CFP+ (n = 5) C57BL/6 mice was determined by flow cytometry. (TIF) [file ppat.1006748.s002.tif]

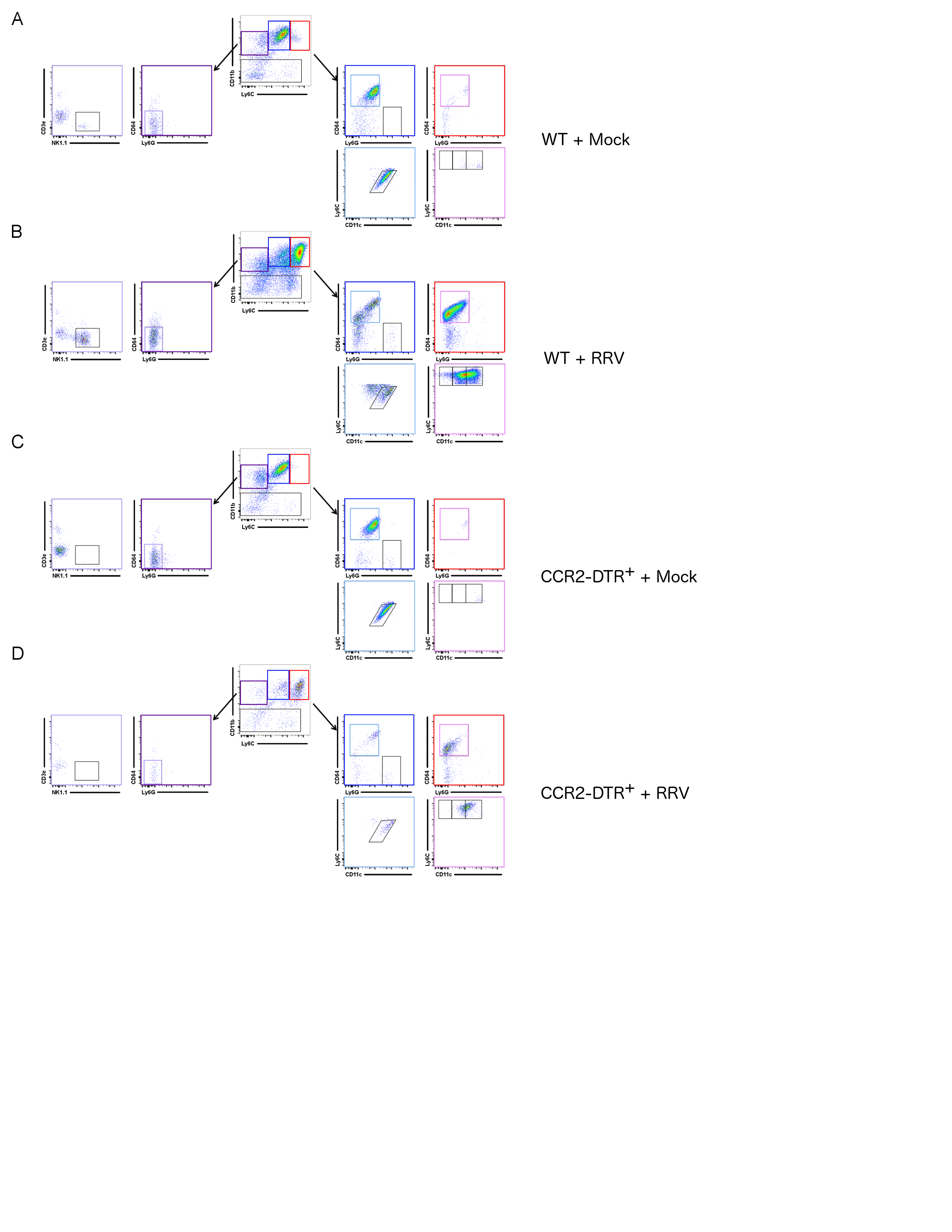

Supplement: S3 Fig — WT (n = 4–5 mice/group) or CCR2-DTR (n = 2–5 mice/group) C57BL/6 mice were inoculated in the left rear footpad with either PBS or RRV-T48. DT was administered at day -1 and day +2 post-inoculation. At 48 h after the last DT administration, the number of NK cells (NK1.1+CD11b+Ly6C-Ly6G-), neutrophils (Ly6G+CD11b+CD43+Ly6C+), and various Ly6C+CD11b+ and Ly6ChiCD11b+ myeloid subsets were determined by flow cytometry using the gating strategy shown. (TIF) [file ppat.1006748.s003.tif]

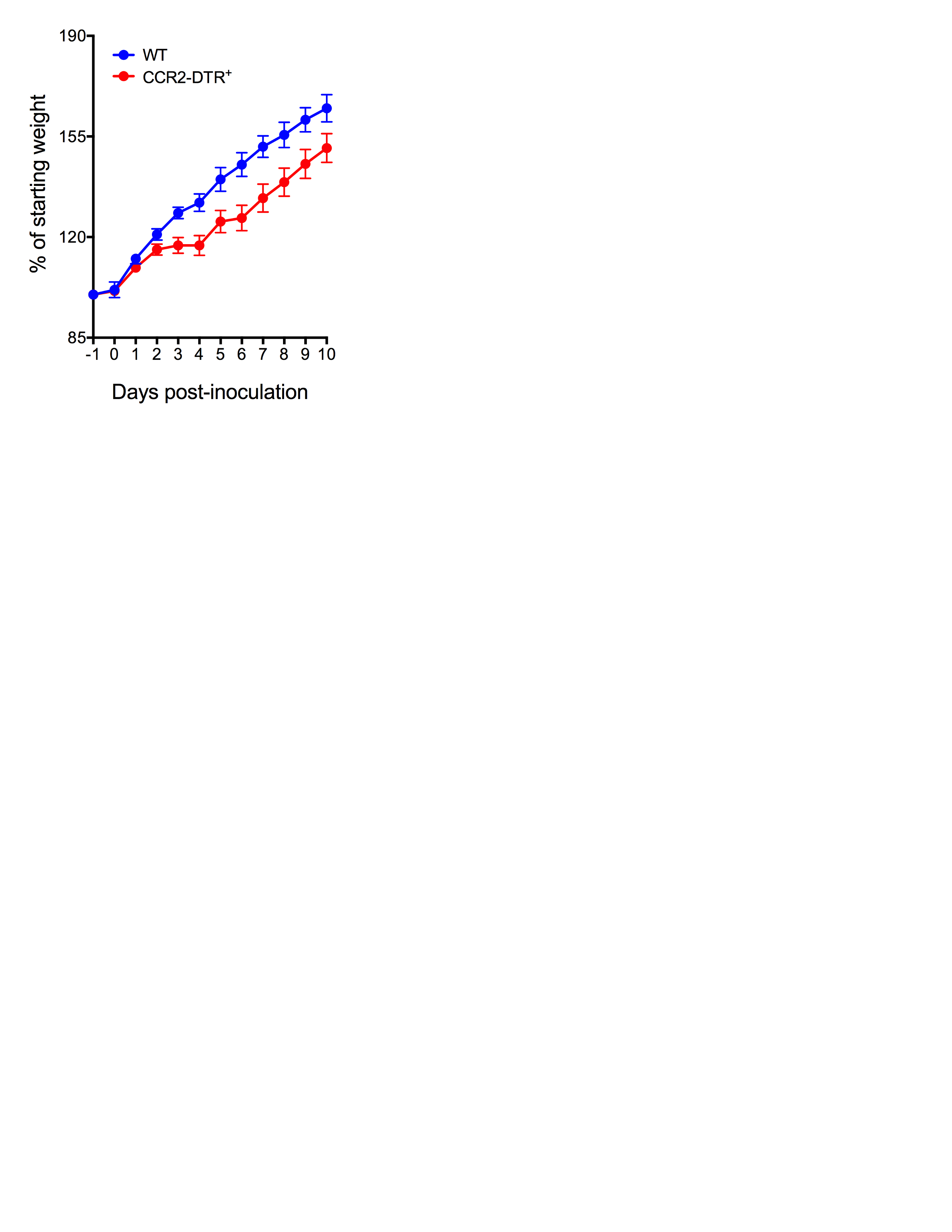

Supplement: S4 Fig — WT (n = 7) or CCR2-DTR+ (n = 10) C57BL/6 mice were inoculated in the left rear footpad with PBS. At days -1 and +2 relative to PBS inoculation, mice were i.p. administered DT. The percent starting body was determined daily. Data are pooled from three independent experiments. (TIF) [file ppat.1006748.s004.tif]

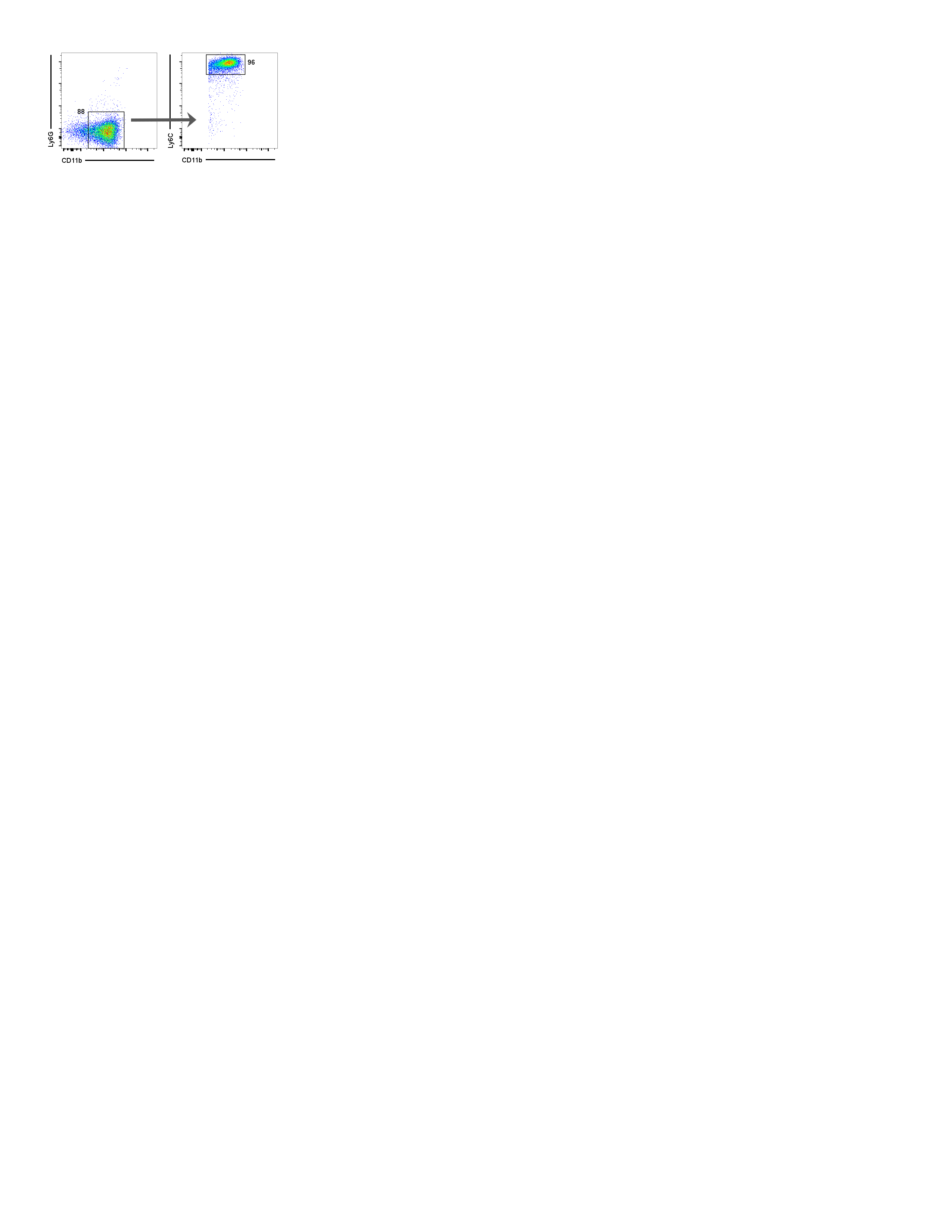

Supplement: S5 Fig — The purity of Ly6Chi monocytes isolated from the bone marrow was assessed by flow cytometry. Cells were incubated with anti-mouse FcγRII/III to block nonspecific antibody binding and then stained with the following antibodies: anti-CD11b (M1/70), anti-Ly6C (HK1.4), and anti-Ly6G (1A8). Shown are representative FACS plots from one of three independent experiments. (TIF) [file ppat.1006748.s005.tif]

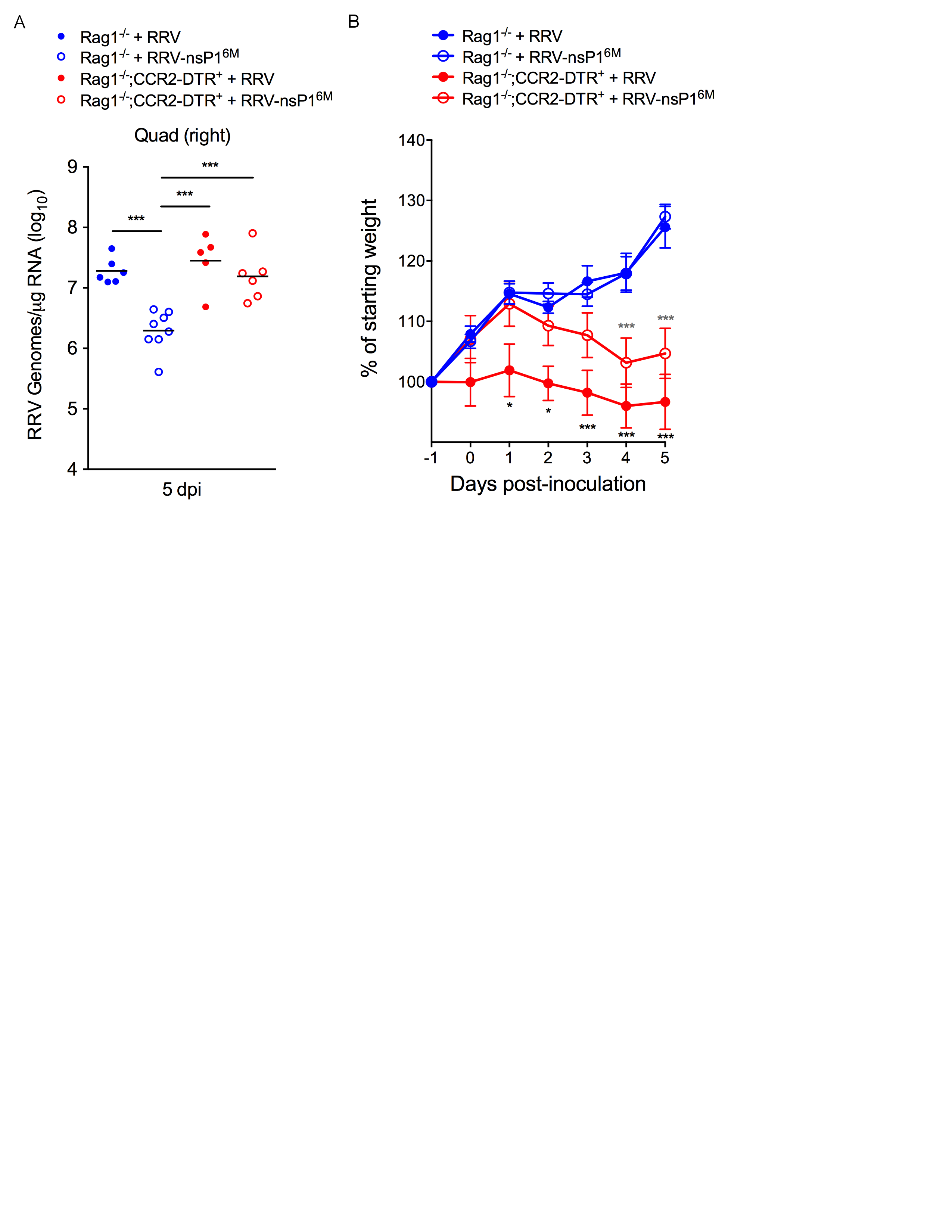

Supplement: S6 Fig — Rag1-/- (n = 6-8/group) or Rag1-/-;CCR2-DTR+ (n = 5-6/group) C57BL/6 mice were inoculated in the left rear footpad with 1,000 PFU of RRV-T48 or RRV-T48-nsP16M. At days -1 and +2 relative to infection, mice were i.p. administered DT. (A) At 5 dpi, viral RNA levels in skeletal muscle tissue were quantified by qRT-PCR. (B) The percent starting body weight was determined daily. Data are pooled from two independent experiments. Statistical comparisons shown are between RRV-T48 in Rag1-/- mice versus Rag1-/-;CCR2-DTR mice (black) and between RRV-T48-nsP16M in Rag1-/- mice versus Rag1-/-;CCR2-DTR mice (gray). P values were determined by one-way ANOVA with a Tukey’s multiple comparison test (A) and a repeated measures two-way ANOVA with a Bonferroni’s multiple comparison test (B).). *, P < 0.05; ***, P < 0.001. (TIF) [file ppat.1006748.s006.tif]

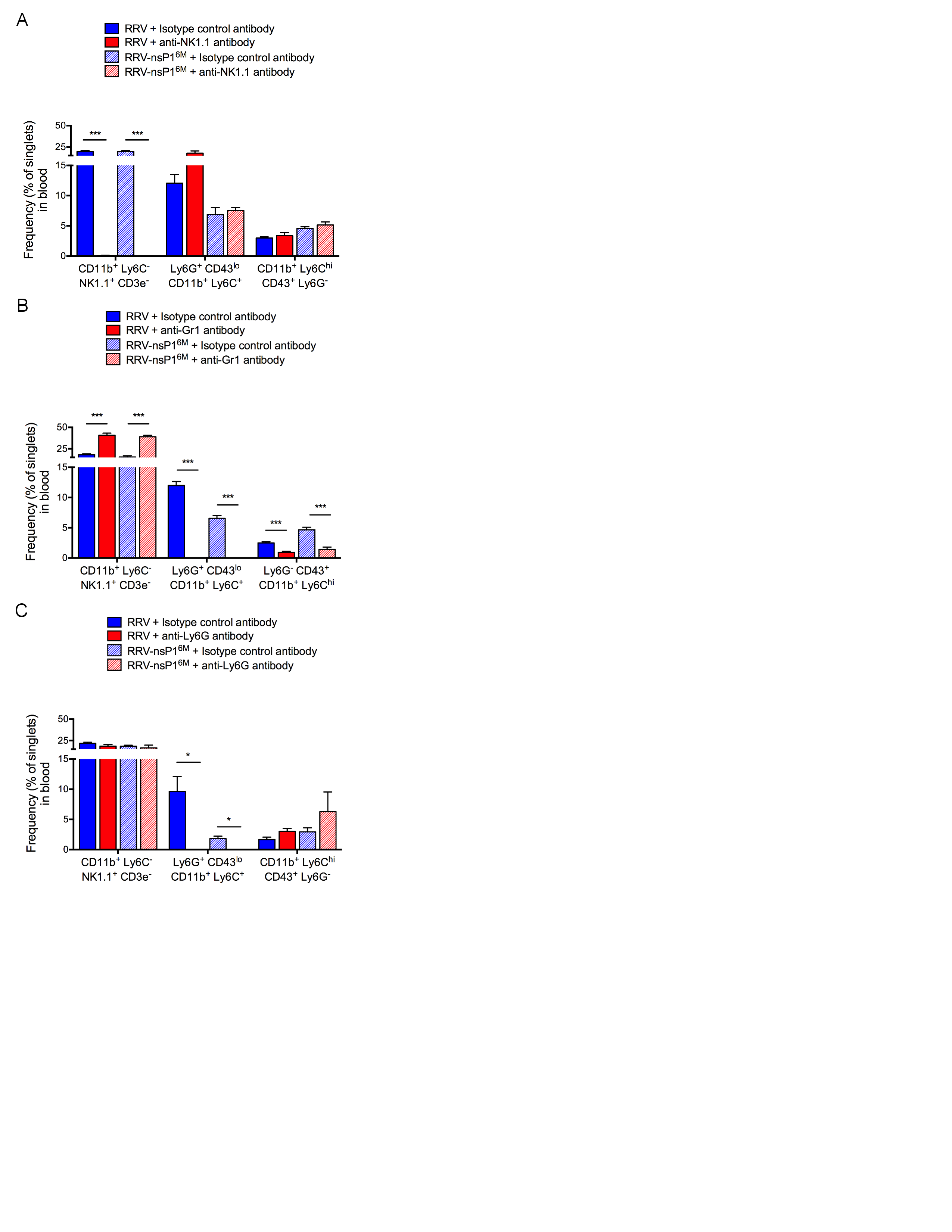

Supplement: S7 Fig — WT C57BL/6 mice were administered (A) anti-NK1.1 (n = 4) or a control antibody (n = 4), (B) anti-Gr1 (n = 8) or a control antibody (n = 8), or (C) anti-Ly6G (n = 4) or a control antibody (n = 4) at day -1 and day +2 relative to infection with the indicated viruses. At 5 dpi, depletion of NK cells, neutrophils, and Ly6Chi monocytes in the circulation was assessed by flow cytometry. (TIF) [file ppat.1006748.s007.tif]

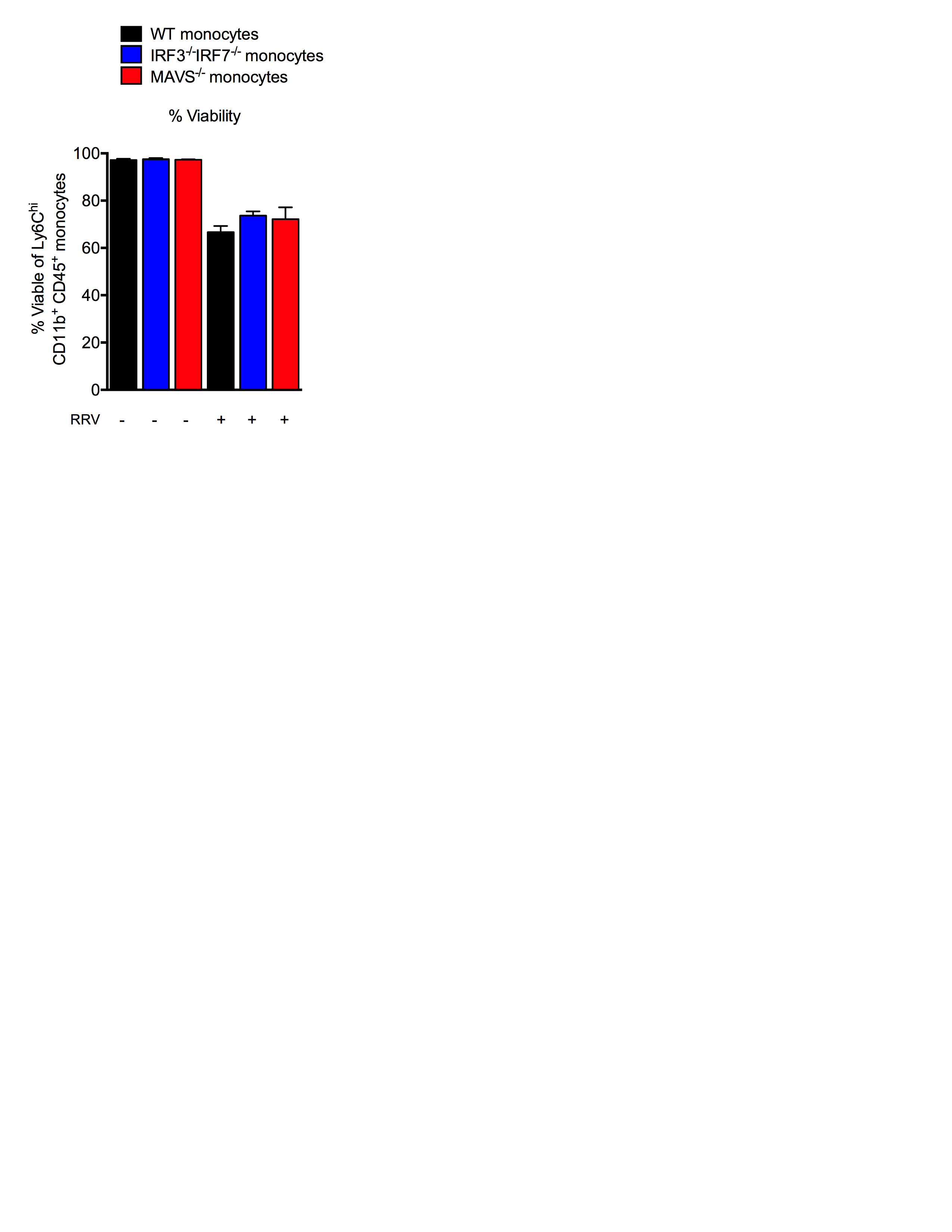

Supplement: S8 Fig — Bone marrow monocytes from WT, Irf3-/-;Irf7-/-, and Mavs-/- mice were co-cultured for 18 h with uninfected or RRV-T48-infected Vero cells. Cells were incubated with LIVE/DEAD Violet Dead Cell Stain, incubated with anti-mouse FcγRII/III to block nonspecific antibody binding, and then stained with the following antibodies: anti-CD11b (M1/70), anti-Ly6C (HK1.4), and anti-CD45 (30-F11). The percent viability of Ly6ChiCD11b+CD45+ cells was determined by flow cytometry (n = 3/group). (TIF) [file ppat.1006748.s008.tif]

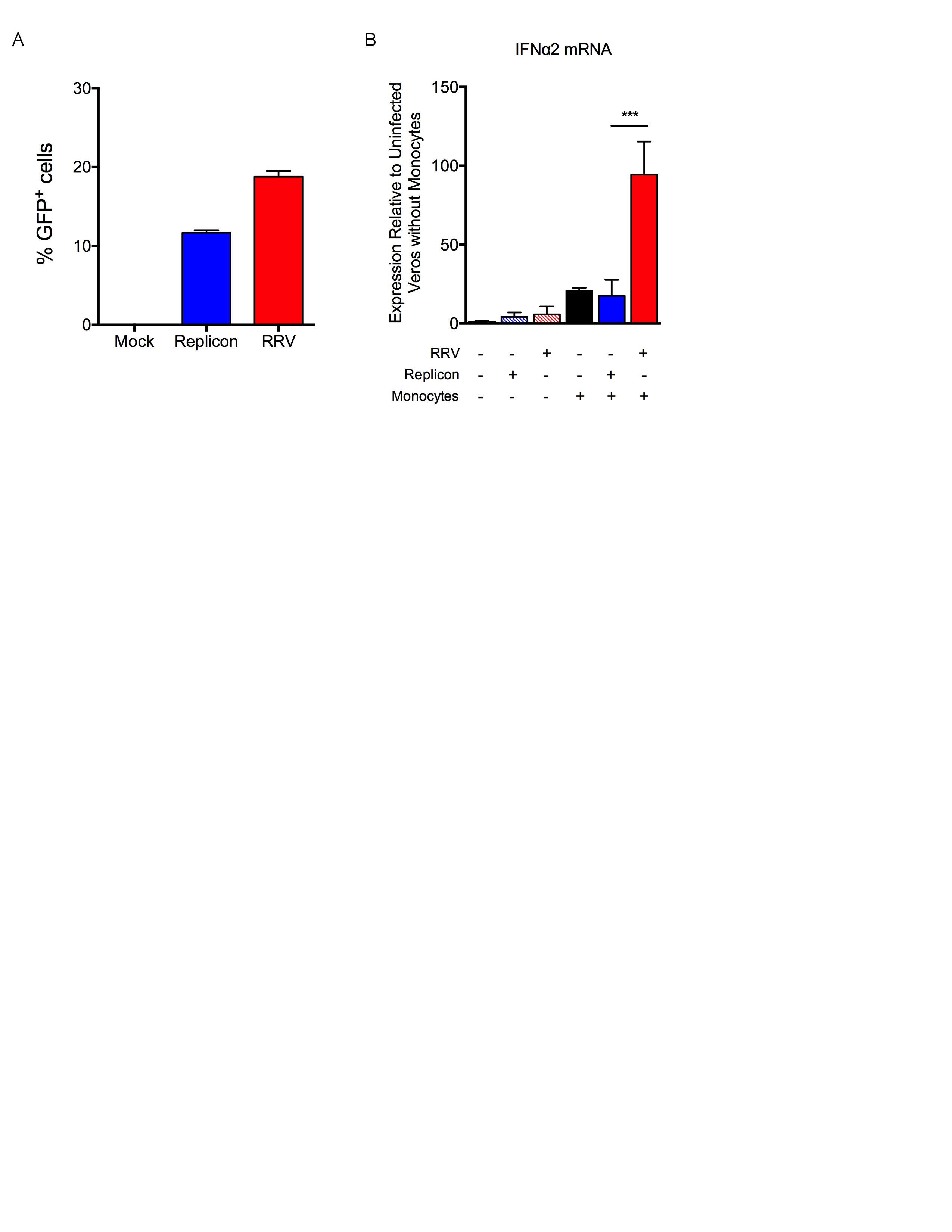

Supplement: S9 Fig — (A-B) Enriched WT bone marrow monocytes were co-cultured with Vero cells electroporated with full-length RRV-GFP RNA or replicon RRV-GFP RNA in which the structural genes are replaced with the GFP gene. After 18 h of co-culture, (A) the percent GFP+ Vero cells was measured by flow cytometry, and (B) IFNα2 mRNA expression level in monocytes was quantified by qRT-PCR. Data are normalized to 18S rRNA levels and are expressed as the relative expression (n-fold increase) over expression in uninfected Vero cells without monocytes. P values were determined by one-way ANOVA with a Tukey’s multiple comparison test. ***, P < 0.001. (TIF) [file ppat.1006748.s009.tif]

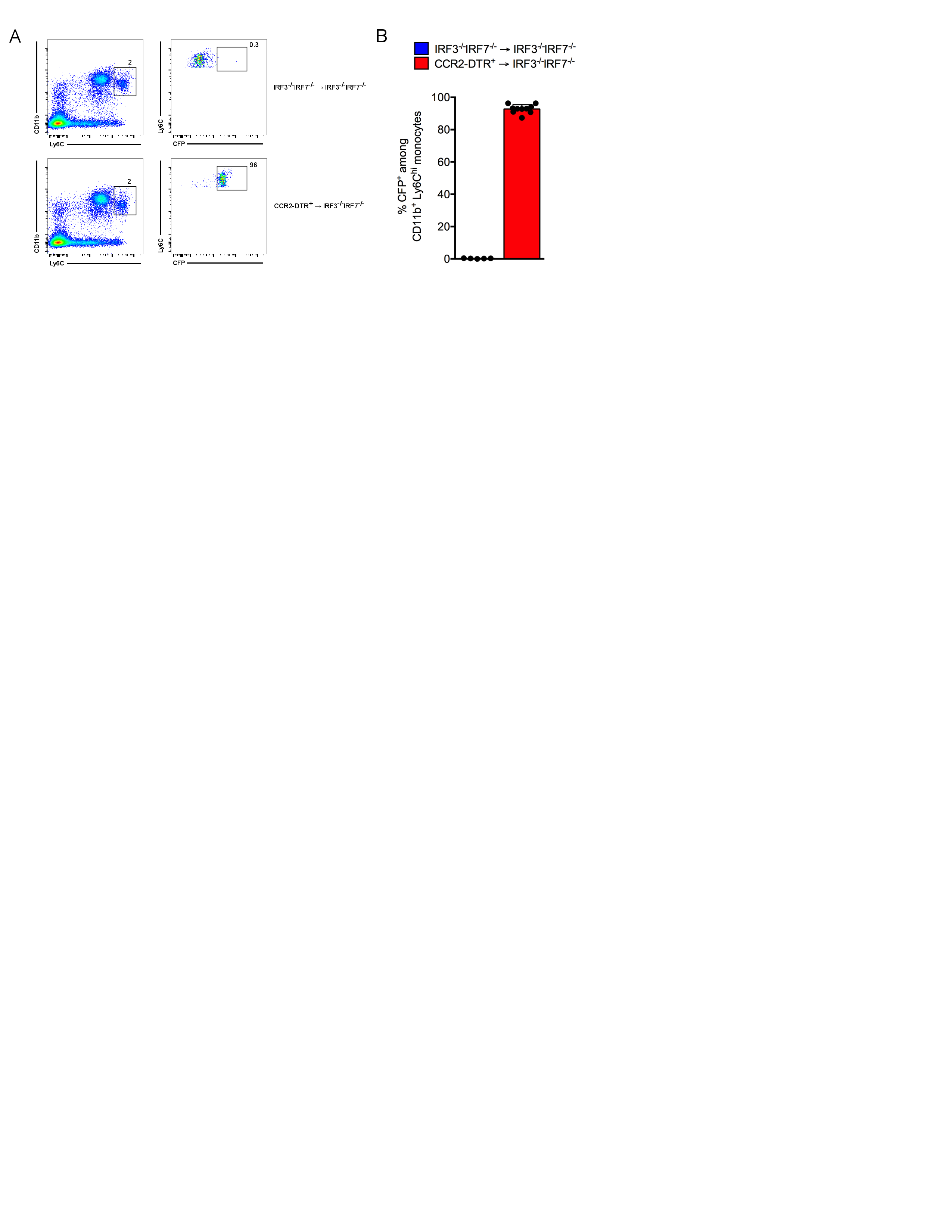

Supplement: S10 Fig — (A-B) Five weeks after reconstitution, successful chimerism in mice receiving CCR2-DTR bone marrow was assessed by measuring CFP expression in circulating CD11b+Ly6Chi monocytes by flow cytometry. (A) The gating scheme used to define CFP expression in Ly6Chi monocytes. (B) Percent CFP expression within the Ly6Chi monocyte gate shown in (A). (TIF) [file ppat.1006748.s010.tif]
